# Mutational and Bioinformatic Analysis of Haloarchaeal Lipobox-Containing Proteins

**DOI:** 10.1155/2010/410975

**Published:** 2010-09-16

**Authors:** Stefanie Storf, Friedhelm Pfeiffer, Kieran Dilks, Zhong Qiang Chen, Saheed Imam, Mechthild Pohlschröder

**Affiliations:** ^1^Department of Biology, University of Pennsylvania, Philadelphia, PA 19104-6018, USA; ^2^Department of Membrane Biochemistry, Max-Planck-Institute of Biochemistry, Martinsried 82152, Germany; ^3^Graduate Group in Genomics and Computational Biology, University of Pennsylvania, Philadelphia, PA 19104-6018, USA; ^4^Program in Cellular and Molecular Biology, University of Wisconsin-Madison, Madison, WI 53706, USA

## Abstract

A conserved lipid-modified cysteine found in a protein motif commonly referred to as a lipobox mediates the membrane anchoring of a subset of proteins transported across the bacterial cytoplasmic membrane via the Sec pathway. Sequenced haloarchaeal genomes encode many putative lipoproteins and recent studies have confirmed the importance of the conserved lipobox cysteine for signal peptide processing of three lipobox-containing proteins in the model archaeon *Haloferax volcanii*. We have extended these *in vivo* analyses to additional *Hfx. volcanii* substrates, supporting our previous *in silico* predictions and confirming the diversity of predicted *Hfx. volcanii* lipoproteins. Moreover, using extensive comparative secretome analyses, we identified genes encodining putative lipoproteins across a wide range of archaeal species. While our *in silico* analyses, supported by *in vivo* data, indicate that most haloarchaeal lipoproteins are Tat substrates, these analyses also predict that many crenarchaeal species lack lipoproteins altogether and that other archaea, such as nonhalophilic euryarchaeal species, transport lipoproteins via the Sec pathway. To facilitate the identification of genes that encode potential haloarchaeal Tat-lipoproteins, we have developed TatLipo, a bioinformatic tool designed to detect lipoboxes in haloarchaeal Tat signal peptides. Our results provide a strong foundation for future studies aimed at identifying components of the archaeal lipoprotein biogenesis pathway.

## 1. Introduction

Most precursors of secreted prokaryotic proteins are transported across cytoplasmic membranes via either the universally conserved Sec pathway or the Twin-arginine translocation (Tat) pathway [1, 2]. The targeting of secreted protein precursors to these translocation pathways is dependent upon the recognition of pathway-specific signal peptides [1, 3]. In bacteria, most substrates transported via these pathways contain a signal peptide processing site that is recognized by signal peptidase I (SPase I) after transfer through the cytoplasmic membrane [3, 4]. However, one type of secreted protein, the bacterial lipoprotein precursors, is processed by signal peptidase II (SPase II), which specifically recognizes a conserved “lipobox” motif at the C-terminus of the signal peptide [4, 5]. The lipobox contains a cysteine residue to which a glyceride-fatty acid lipid is attached by a prolipoprotein diacylglyceryl transferase (Lgt) [6, 7]. SPase II cleaves the precursor immediately upstream of this lipid-modified cysteine. In Gram-negative and some Gram-positive bacteria, the conserved lipobox cysteine residue is also acylated by apolipoprotein *N*-acyltransferase [8, 9].

Sequence analyses of genomes isolated from archaea that thrive in high salt environments have identified a large number of open reading frames that encode putative Tat substrates containing potential lipoboxes [10–13]. Mass spectrometry results of the haloalkaliphilic halocyanin from *Natronomonas pharaonis *was consistent with the presence of an N-terminal cysteine residue modified by a lipid consisting of two C20 phytanyl groups linked to a glycerol group and also being acetylated at the amino group [13].

When the conserved lipobox cysteine residue is replaced with a serine residue, putative *Hfx. volcanii* lipoprotein precursors are not processed, suggesting that these haloarchaeal Tat substrates are in fact lipoproteins [12]. Interestingly, while unprocessed precursors of similar replacement mutants remain cell-associated in bacteria, the three mutant *Hfx. volcanii* precursors thus far tested are secreted into the supernatant [12]. Homologs of bacterial lipid-modification enzymes have not been detected in archaeal genomes. Considering this, release of the unprocessed mutant constructs into the extracellular environment supports the hypothesis that the molecular mechanisms underlying archaeal lipoprotein biosynthesis are distinct from their bacterial counterparts. It should also be noted that recently published data has revealed that some Gram-positive bacterial lipoproteins are also Tat substrates [14, 15]; however, little is known about the lipid-anchoring process in these bacteria. One possible interpretation of this result was that the release of the haloarchaeal Tat precursor proteins into the supernatant might reflect a difference in the mechanisms involved in lipid-modification of Sec and Tat substrates. 

In this study, we carried out additional *in vivo* and *in silico* analyses to gain further insight into the processes involved in archaeal Tat and Sec lipoprotein biosynthesis and the diversity of archaeal lipoproteins. Although replacing the lipobox cysteine of additional putative Tat lipoproteins with a serine blocked precursor processing, these unprocessed mutant proteins remained membrane associated, indicating that secretion of unprocessed cysteine to serine replacement mutants is not a universal phenomenon, for either archaeal lipoprotein precursors or for Tat substrate lipoproteins. Conversely, complementary *in silico* analyses suggest that an extensive use of lipid-anchoring by membrane-associated Tat substrates is restricted to haloarchaea while it appears to be rare, or even non-existent, in other archaeal phyla. To identify genes that encode putative lipoprotein precursors, we employed existing lipoprotein prediction programs primarily trained on sets of bacterial Sec lipoproteins. This allowed us to compile a set of 484 putative lipoproteins from six haloarchaeal genomes. These data were used to develop a novel Tat-specific lipoprotein prediction tool for halophilic archaea.

## 2. Materials and Methods

### 2.1. Reagents

All enzymes used in standard molecular biology procedures were purchased from New England Biolabs, except for *iProof *High-Fidelity DNA Polymerase, which was purchased from Biorad. The ECL Plus Western blotting system, horseradish peroxidase-linked sheep anti-mouse antibody was purchased from Amersham Biosciences. The anti-myc monoclonal antibody and polyvinylidene difluoride membrane were purchased from Millipore. DNA and plasmid purification kits, and the anti-Penta-His antibody were purchased from QIAGEN. NuPAGE gels, buffers, and reagents were purchased from Invitrogen. Difco agar and Bacto yeast extract were purchased from Becton, Dickinson and Company. Peptone was purchased from Oxoid. All other chemicals and reagents were purchased from either Fisher or Sigma.

### 2.2. Strains and Growth Conditions

The plasmids and strains used in this study are listed in Supplementary Table 1 (available online at doi:10.1155/2010/410975). *Hfx. volcanii* strains were routinely grown at 45°C in 18% MGM [16]. MGM was supplemented with novobiocin (2.0 *μ*g ml^−1^) and thymidine (40 *μ*g ml^−1^), when necessary. *Escherichia coli* strains were grown at 37°C in NZCYM and supplemented with ampicillin (100 *μ*g ml^−1^).

### 2.3. Construction of Expression Vectors

The Hvo_*B0139*, Hvo_*1242*, Hvo_*1808*, Hvo_*1609*, and Hvo_*1580 *genes were placed under the control of *P_fdx_* [17] and cloned into pMLH3 [18] with a C-terminal Myc tag generating plasmids listed in Supplementary Table 1. *P_fdx_* was amplified from pGB70 using the FdxFor and FdxRev primers, and the substrate genes were amplified using the relevant primers (see Supplementary Table 2). Substrate genes were then placed under the control of *P_fdx_* by overlap PCR, using both the substrate genes and the *fdx* promoter fragment PCR products as template. The resulting *P_fdx_*, *P_fdx_-B0139*, *P_fdx_-1242*, *P_fdx_-1808*, *P_fdx_-1609*, and *P_fdx_-1580* fragments were digested with BamHI and HindIII, and ligated into pMLH3 that had been digested with BamHI and HindIII and treated with Calf Intestinal Phosphatase. The sequence of the construct inserts was confirmed by DNA sequencing.

In addition to the listed Myc-tagged wild-type substrate constructs, several mutant constructs were generated. In all cases, wild-type substrate constructs were used as DNA templates for PCRs. The twin-arginine residues of these gene products were altered to twin lysines via overlap PCR, using the above constructs as DNA template and primers that resulted in replacement of the arginine codons with lysine codons (AAG) (Supplementary Table 2). For the genes encoding putative lipoprotein substrates, likely lipobox cysteine codons were altered to serine codons via overlap PCR, using primers that resulted in replacement of the cysteine codon with a serine codon (TCG) (Supplementary Table 2). For signal sequence deletion mutants, the potential signal sequence located prior to the N-terminal cysteine was deleted via PCR using primers listed in Supplementary Table 2. The fidelity of these mutant constructs was confirmed by DNA sequencing. Similar methods were used to clone Hvo_*0494* and Hvo_*0494C20S*, except that these constructs were cloned into pRV1-p*tna* plasmid under the control of the tryptophan-inducible promoter p*tna* [19]. Genes were amplified using the primers listed in Supplementary Table 2 and inserted into pRV1-p*tna* digested with NdeI and EcoRI.

Wild-type and mutant constructs were extracted from DH5*α* (Invitrogen) and passed through DL739 [20] to obtain nonmethylated DNA, which was subsequently transformed into *Hfx. volcanii* strain H99 [21] or KD5 [22] using the standard PEG method [16].

### 2.4. Expression and Localization of Tat Substrates

Liquid cultures of relevant strains were grown until mid-log (OD_600_ ~ 0.5). Subsequently, cells were collected by centrifugation at 4300 g for 10 min at 4°C. The supernatants were recentrifuged at 4300 g for 10 min to remove cellular contamination, and secreted proteins were precipitated with cold TCA (100% v/v) then washed twice with cold acetone (80% v/v). The cell pellets were washed once with MGM then pelleted again as described and resuspended in 1× NuPAGE lithium dodecyl sulfate (LDS) Sample buffer.

### 2.5. Immunoblotting

All protein samples were stored at −20°C in 1× NuPAGE LDS sample buffer supplemented with 50 mM dithiothreitol (DTT). Samples were run on Bis-Tris NuPAGE gels under denaturing conditions using either morpholinepropanesulfonic acid (MOPS) or 2-(N-morpholino)ethanesulfonic acid (MES) running buffer, or on Tris-Acetate NuPAGE gels using TA running buffer. Proteins were transferred to polyvinylidene difluoride (PVDF) membrane using the Bio-Rad Transblot-SD Semi-Dry Transfer Cell at 15 V for 30 minutes. Three buffers were used in semidry transfer: anode I [300 mM Tris, 10% (v/v) methanol, pH 10.4], anode II [25 mM Tris, 10% (v/v) methanol, pH 10.4], and cathode [25 mM Tris, 40 mM glycine, and 10% (v/v) methanol, pH 9.4]. PVDF membranes were probed with the primary antibodies anti-Penta-His (1:1000) or anti-Myc (1:1000) and secondary anti-mouse antibody (1:10,000). All Western blots shown are representative of at least two independent experiments.

### 2.6. Archaeal Secretome Analyses

Various commonly available bioinformatic tools were used to analyze the secreted proteins of six haloarchaeal species, nine non-halophilic euryarchaeal species, nine crenarchaeal species, as well as three other archaeal species (see Supplementary Table 3). For lipoprotein predictions, we used three independent prediction programs: (i) the Prosite position-specific matrix PS51257 (PROKAR_LIPOPROTEIN) [23]; (ii) LipoP [24]; and (iii) predLipo [25]. To keep false positive predictions minimal, candidate proteins are considered lipobox-positive only when they are recognized by at least two of these predictors (also see Supplementary Text and Supplementary Figure 1). To identify Tat substrates, we used TatFind [26]. Predicted Tat substrates that were not predicted to be lipoproteins were designated Tat substrates with an SPase I cleavage site in their signal peptides. All predicted lipoproteins not identified by TatFind were designated Sec substrates with signal peptides processed by SPase II. 

Phobius [27] was used to identify Sec substrate signal peptides cleaved by SPase I. However, we used caution when considering this data, as there is a strong tendency for Phobius to predict false positives for both Sec signal peptides and SPase I cleavage sites. Since both the Sec and Tat pathway-specific signal peptides contain a charged region followed by a hydrophobic core, and many contain an SPase I cleavage site, Phobius is inclined to misclassify many Tat signal peptides as Sec signal peptides [28]. Consequently, we disregarded Phobius predictions of Sec signal peptides in TatFind positives. Similarly, since SPase I has a relaxed specificity, which therefore necessitates relaxed cleavage site prediction constraints, many lipoproteins cleaved by SPase II are predicted to have signal peptides processed by SPase I by Phobius. Consequently, we disregarded Phobius predictions of SPase I cleavage sites for predicted lipoproteins, as predicted by the method described above (also see Supplementary Figure 1).

### 2.7. Tat Lipoprotein Prediction

To extract position-specific amino acid statistics, amino acid sequences of 484 lipoproteins predicted from six haloarchaeal genomes were “aligned” as follows: for 400 lipoproteins predicted to be Tat substrates, the sequences of the lipobox and the Tat motif, as predicted by the lipoprotein prediction programs and TatFind, respectively, were aligned. These alignments were attained by introducing a gap of variable length between the fifth and sixth amino acid residue after the second arginine of the twin arginine motif.

The lipoprotein set contains 50 TatFind negative sequences that contain two consecutive arginines in the charged region of the signal peptide. However, specific amino acid residues at positions +1, +4, +5, and/or +6, relative to these arginines are not allowed by TatFind. Some of these sequences may be false negatives caused by the stringent rules applied by TatFind. Therefore, the twin arginines in these potential false negatives were aligned with the twin arginines in the Tat motifs of the TatFind positives.

Most of the 34 remaining sequences that were examined were found to contain a single arginine. We aligned this arginine with the second arginine of the twin arginines of the Tat motifs identified by TatFind unless the preceding residue was the initiator methionine, in which case we aligned this arginine with the first arginine of the Tat motifs (see Supplementary Table 4).

The 484 aligned haloarchaeal lipoproteins (Supplementary Table 4) were used to compute position-specific amino acid frequencies. These frequencies were used to develop an algorithm using a rule-based approach to detect haloarchaeal lipobox motifs. For the positions with the strongest composition bias, amino acids were categorized as “required”, “frequent”, “normal”, “tolerated”, or “excluded”. Required and excluded amino acids were used in regular expressions, that is, no exceptions were allowed. Tolerated amino acids are not excluded, although they have not been found in the set of 484 aligned haloarchaeal lipoproteins at the corresponding position. However, they are similar to other amino acids, which have been found (e.g., tyrosine or tryptophane in positions where phenylalanine is found) (see also Supplementary Table 5). Moreover, tolerated amino acid residues are allowed only when other positions are occupied by frequent amino acids. The rule-based algorithm is the basis of TatLipo (available at SignalFind.org), which is an extension of TatFind, a program which identifies haloarchaeal Tat substrates with a high degree of accuracy [26, 29].

## 3. Results

### 3.1. Some Unprocessed*Hfx. volcanii* Lipobox Replacement Mutant Proteins Remain Cell-Associated

**Figure 1:**
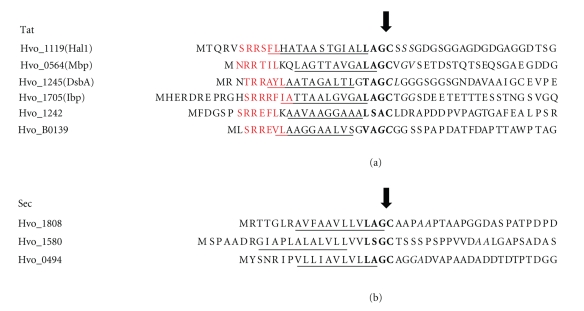
Tat and Sec signal peptides containing lipobox motifs. N-terminal regions of the precursors of (a) Tat; and (b) Sec substrates with lipoboxes (bold) predicted by at least two of the three lipoprotein prediction programs (PredLipo, LipoP and Prosite PS51257), with the exception of Hvo_1242, which was only LipoP-positive. Tat motifs (red) were predicted by TatFind, hydrophobic stretches (underlined), and SPase I cleavage sites (*italics*) were predicted by Phobius. An arrow indicates the predicted SPase II cleavage sites.

**Figure 2:**
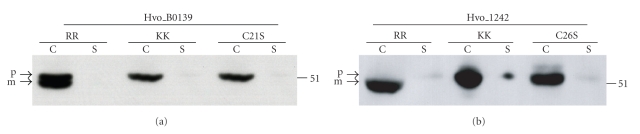
*Hfx. volcanii* proteins Hvo_B0139 and Hvo_1242 are Tat substrates that require the lipobox cysteine for processing but not for anchoring to the cytoplasmic membrane. Western blot analyses of the wild-type (RR), twin lysine replacement mutants (KK), and cysteine to serine replacement mutants (C21S and C26S for Hvo_B0139, and Hvo_1242, resp.). All proteins expressed had C-terminal Myc-tags and were detected using anti-Myc antibodies. Comparable amounts of protein were loaded in each lane. The migration of molecular weight standards is indicated on the right. Predicted positions of precursor (p) and mature (m) proteins are indicated.

To further investigate the diversity of haloarchaeal lipoproteins secreted via the Tat pathway, we chose to characterize two additional proteins with distinct lipobox motifs, Hvo_B0139 (VAGC), predicted by three lipoprotein prediction programs (Prosite, predLipo and LipoP) and Hvo_1242 (LSGC), which was only predicted by LipoP (Figure 1(a) and see below). Consistent with these putative Tat substrates being lipoproteins, we determined that both are cell-associated (Figures 2(a) and 2(b)). Protein extracts isolated from *Hfx. volcanii* that express C-terminally Myc-tagged versions of these proteins were subjected to denaturing polyacrylamide gel electrophoresis (PAGE), followed by Western blot analyses. When genes are overexpressed from a plasmid, incomplete processing of the encoded protein is frequently observed. Consistent with the presence of a precursor and a processed protein, anti-Myc antibody detected two distinct protein bands in Western blots of extracts containing the Myc-tagged version of Hvo_B0139. Only a single protein band was detected in extracts containing Myc-tagged Hvo_1242. To provide evidence that these proteins are Tat substrates, we mutated the essential twin arginines that have been shown to be essential for protein export by the Tat translocase. Mutant proteins in which twin arginine residues in the signal peptide were replaced with twin lysines (Hvo_B0139KK and Hvo_1242KK, resp.) were overexpressed in wild-type *Hfx. volcanii*, and protein extracts containing these mutant proteins were subjected to denaturing PAGE, followed by Western blot analyses. Hvo_B0139KK migrated as a single protein band, at a position on the gel similar to that of the Hvo_B0139 precursor protein (Figure 2(a)). Moreover, Western blots of protein fractions isolated from *Hfx. volcanii* that overexpress Hvo_1242KK detected one protein band, which migrates at a slower rate than Hvo_1242, indicating that this mutant protein is not processed (Figure 2(b)). 

To determine the importance of putative archaeal protein lipoboxes in signal peptide processing and membrane-anchoring, we replaced the conserved lipobox cysteines in these archaeal Tat substrates with serines. Western blot analysis of purified protein extracts subjected to denaturing PAGE showed that Hvo_B0139C21S and Hvo_1242C26S replacement mutants migrate at a position on the gel similar to that of the corresponding precursor, indicating that the cysteine is required for signal peptidase processing (Figures 2(a) and 2(b)). Interestingly, although Giménez et al. showed that DsbA, Mbp, and Ibp cysteine to serine replacement mutants are released into the supernatant [12], the precursor forms of Hvo_B0139C21S and Hvo_1242C26S remain cell associated, which might be due to the unprocessed signal serving as a membrane anchor (Figure 2).

### 3.2. The Transport of Lipobox-Containing Proteins Is Not Dependent on a Particular *Hfx. volcanii* TatA Paralog

**Figure 3:**
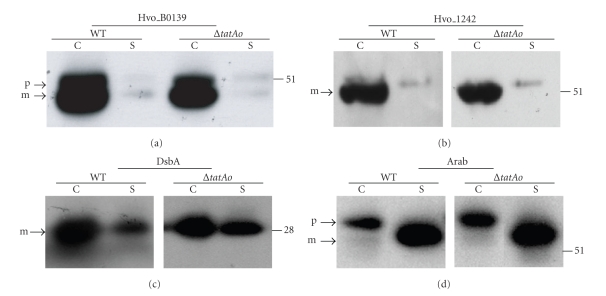
Putative Tat substrate lipoproteins Hvo_B0139, Hvo_1242, DsbA and arabinanase are translocated independent of TatAo in* Hfx. volcanii*. Western blot analyses of wild-type (RR) Hvo_B0139, Hvo_1242, and DsbA (Hvo_1245) and arabinanase (Hvo_B0232) expressed in *Hfx. volcanii* wild-type (WT) or TatAo deletion mutants (∆*tatAo*). All proteins expressed had C-terminal Myc-tags, and were detected using anti-Myc antibodies. Comparable amounts of protein were loaded in each lane. The migration of molecular weight standards is indicated on the right. Predicted positions of precursor (p) and mature (m) proteins are indicated. For comparisons of the unprocessed and processed substrate migration see Figure 2.

Although TatAt is essential for *Hfx. volcanii* survival under standard laboratory conditions, TatAo, the *Hfx. volcanii* paralog of TatAt, is not [22]. To determine whether the membrane association of unprocessed cysteine to serine replacement mutants transported via the Tat pathway depends upon a TatA paralog distinct from that involved in transporting similar mutants that are released into the extracellular environment, we investigated the transport of the Hvo_B0139 and Hvo_1242, as well as DsbA, in *Hfx. volcanii* ∆*tatAo *mutants. We determined that these lipobox-containing proteins are transported with similar efficiencies in wild-type cells and ∆*tatAo* mutants (Figures 3(a)–3(c)). Since the soluble Tat substrate arabinanase is also secreted in a TatAo-independent manner (Figure 3(d)), it is clear that in *Hfx. volcanii* the TatAt role in transport is not limited to the secretion of lipoproteins. 

### 3.3. Putative *Hfx. volcanii* Sec Substrate Lipobox C-S Replacement Mutants Are Processed, but Are Relatively Unstable

**Figure 4:**
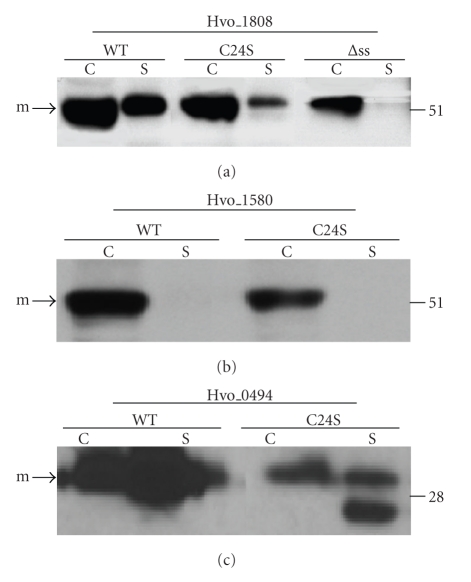
Cysteine to serine mutants of Hvo_1808, Hvo_1580, and Hvo_0494 are less stable than wild-type constructs but appear to be processed. Western blot analyses of wild-type proteins (WT), cysteine to serine replacement mutants (Hvo_1808C19S, Hvo_1580C24S and Hvo_0494C20S), and signal sequence deletion mutants (∆ss) of Hvo_1808. All proteins expressed had C-terminal Myc-tags except for Hvo_0494 and Hvo_0494C20S, which were C-terminally His-tagged. Myc and His-tagged proteins were detected using anti-Myc and anti-His antibodies, respectively. Comparable amounts of protein were loaded in each lane. The migration of molecular weight standards is indicated on the right. Predicted positions of precursor (p) and mature (m) proteins are indicated.

To determine whether archaeal Sec substrates containing lipobox motifs require the conserved cysteine for processing, we characterized three putative *Hfx. vocanii* Sec substrates with potential lipoboxes: Hvo_1808 (LAGC), Hvo_1580 (LSGC), and Hvo_0494 (LAGC). Consistent with Hvo_1808 and Hvo_1580 being lipoproteins, they are primarily retained in cell-associated protein fractions (Figures 4(a) and 4(b)). Although the supernatant fraction of Hvo_1808 expressing cultures contained a minor protein band that migrated with one of the bands seen in the membrane-associated fraction, it is likely that this results from protein shedding during the isolation of protein fractions, as is the case for many bacterial lipoproteins (also observed for the *Hfx. volcanii* DsbA, [12]). Shedding may also be the reason why a large portion of Hvo_0494 is found in the supernatant fraction (Figure 4(c)). To confirm that these Sec substrates are processed, we attempted to overexpress signal peptide deletion mutants of these three proteins (Hvo_1808Δss, Hvo_1580Δss, and Hvo_0494Δss), which should have the same molecular weights as the processed proteins. Although Hvo_1508Δss and Hvo_0494Δss appear to be unstable, as no corresponding protein was identified by Western blot analysis (data not shown), Hvo_1808Δss migrated at the same position on the gel as the wild-type protein when separated by denaturing PAGE, indicating that the wild-type protein is processed (Figure 4(a)). 

Interestingly, when the conserved lipobox cysteine in these Sec substrates was replaced with a serine, Western blot analysis showed that, when separated by denaturing PAGE, Hvo_1808C19S and Hvo_1580C24S migrate at the same position on the gel as the wild-type proteins and are found in the same relative amounts in the cell-associated and supernatant protein fractions, indicating that neither the processing nor the membrane anchoring of these mutant proteins is dependent on the lipobox cysteine (Figures 4(a) and 4(b)). In fact, all three Sec substrates also contain potential SPase I processing sites, as predicted by Phobius [27] (Figure 1(b)). However, Western blot analyses also revealed that all three mutant proteins are less abundant than the corresponding wild-type proteins, in both the cell associated and the supernatant protein fractions, suggesting that these replacement mutants are less stable than the wild-type proteins. In fact, in addition to the mutant proteins being less abundant than the wild-type proteins, Western blot analysis of protein fractions containing Hvo_0494C20S revealed a faster migrating band in the supernatant fraction, possibly a degradation product (Figure 4(c)).

### 3.4. Prediction of Archaeal Lipoproteins

Upon determining that many predicted *Hfx. volcanii *Tat signal peptides contain putative lipoboxes, and confirming that the conserved lipobox cysteine in these substrates is important for precursor processing, we resolved to determine (i) whether Tat substrates of other haloarchaea frequently contain SPase II processed signal peptides—as has been suggested for at least two additional haloarchaea species [10, 11]; (ii) whether non-halophilic archaeal genomes also encode predicted lipoproteins; and (iii) whether these putative non-halophilic lipoproteins are typically predicted Sec or Tat substrates. 

As there was no archaeal-specific lipoprotein prediction program available, we used other existing tools: (i) the Prosite position-specific matrix PS51257 (PROKAR_LIPOPROTEIN) [23] and LipoP [24], which have both been mainly trained on lipoproteins encoded by Gram-negative bacterial genomes; and (ii) predLipo [25], which has been trained on lipoproteins encoded by Gram-positive bacterial genomes. The results of these three predictors did not correlate well for archaeal genomes (see the Supplementary Text). Only 43% of the initial predictions were common to all three programs, while 24% were specific to only one of them. To increase the reliability of the prediction, we requested that at least two of the three prediction programs must be positive for a protein to consider it a lipoprotein (i.e., as having an SPase II cleavage site). Excluding results obtained with only a single predictor resulted in a set of 484 predicted lipoproteins from six halophilic archaea (Supplementary Table 4). Of these, 56% were positive by all three predictors and 44% were positive by only two of the three predictors. In the following, we refer to these as lipoproteins although experimental confirmation in archaea is still lacking except for halocyanin from *N. pharaonis *[13]. However, we consider the requirement for positive results from two independent predictors a strong constraint so that we expect only a small fraction will be false positives.

The lipoproteins predicted by this method were used in two ways. Predictions for a total of 27 archaeal genomes were used for statistical analyses. The set of 484 haloarchaeal lipoproteins was used to determine position-specific amino acid frequencies, results of which were used to develop TatLipo, a haloarchaea-specific prediction program for lipoproteins that are secreted by the Tat pathway.

### 3.5. Extensive Anchoring of Tat Substrates via Lipid Anchor Appears to Be Unique to Haloarchaea

**Figure 5:**
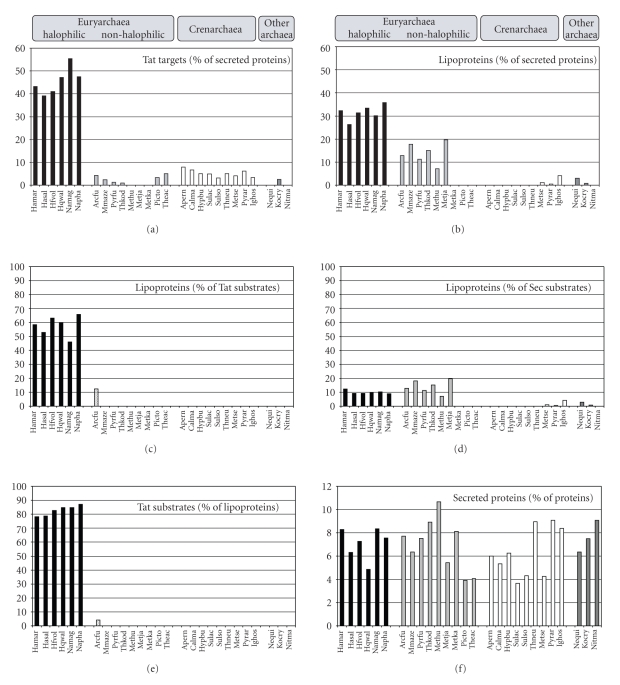
Only haloarchaeal Tat substrates are predicted to be predominantly lipoproteins. The predicted relative proportions are shown for several types of secreted proteins for halophilic and non-halophilic euryarchaeal species as well as crenarchaeal species and three species belonging to other archaeal phyla. (a) The percentage of secreted proteins that are Tat substrates. (b) The percentage of secreted proteins that are lipoproteins. (c) The percentage of Tat substrates that are lipoproteins. (d) The percentage of Sec substrates that are lipoproteins. (e) The percentage of lipoproteins that are secreted via the Tat pathway. (f) The percentage of proteins that are secreted (predicted Tat and Sec substrates with SPase I or SPase II cleavae sites). Raw data are available in Supplementary Table 4. Organisms are abbreviated as follows: haloarchaea (Hamar: *Haloarcula marismortui*; Hasal: *Halobacterium salinarum*; Hfvol: *Haloferax volcanii*; Hqwal: *Haloquadratum walsbyi*; Namag: *Natrialba magadii*; Napha: *Natronomonas pharaonis*), other euryarchaea (Arcfu: *Archaeoglobus fulgidus*; Mmaze: *Methanosarcina mazei*; Pyrfu: *Pyrococcus furiosus*; Thkod: *Thermococcus kodakarensis*; Methu: *Methanospirillum hungatei*; Metja: *Methanocaldococcus jannaschii*; Metka: *Methanopyrus kandleri*; Picto: *Picrophilus torridus*; Theac: *Thermoplasma acidophilum*), crenarchaea (Apern: *Aeropyrum pernix*; Calma: *Caldivirga maquilingensis*; Hypbu: *Hyperthermus butylicus*; Sulac: *Sulfolobus acidocaldarius*; Sulso: *Sulfolobus solfataricus*; Thneu: *Thermoproteus neutrophilus*; Metse: *Metallosphaera sedula*; Pyrar: *Pyrobaculum arsenaticum*; Ighos: *Ignicoccus hospitalis*), and other archaea (Nequi: *Nanoarchaeum equitans*; Kocry: *Korarchaeum cryptofilum*; Nitma: *Nitrosopumilus maritimus*).

Consistent with previous analyses, we determined that 39–55% of the haloarchaeal proteins are secreted via the Tat pathway (Figure 5(a)). However, although most archaea appear to secrete some of their proteins via the Tat pathway, Tat substrates account for no more than 8% of any non-halophilic archaeal secretome thus far analyzed (Figure 5(a), Supplementary Table 3) [29]. The lack of predicted Tat substrates in the euryarchaea *M. hungatei, M. jannaschii, *and* M. kandleri*, or in *N. equitans* and* N. maritimus*, is consistent with the absence of homologs of Tat pathway components in these organisms ([29] and data not shown).

Conversely, while proteins that contain signal peptides with lipobox motifs are common in some euryarchaea, we found that they are absent in many crenarchaea (Figure 5(b), Supplementary Table 3). In the euryarchaea, the fraction of predicted lipoproteins is somewhat higher among haloarchaea (26–36%) than it is among non-halophilic species (7–20%). Six of the nine analyzed crenarchaeota completely lack predicted lipoproteins. *Ignicoccus hospitalis,* the only archaeon known to have an outer membrane [30]), is an unusual crenarchaeon, having a secretome in which 4.2% of the proteins have a lipobox-containing signal peptide (Figure 5(b), Supplementary Table 3). 

Although roughly half of the haloarchaeal Tat substrates contain a lipobox motif (46–65%) (Figure 5(c), Supplementary Table 3), we determined that only about 10% of haloarchaeal Sec substrates contain one (Figure 5(d), Supplementary Table 3). However, while haloarchaea are unique among archaea with respect to the portion of secreted proteins containing the Tat/lipobox combination, they are relatively similar to other euryarchaea in regard to the frequency of secreted proteins having the Sec/lipobox combination (Figure 5(d)).

Furthermore, a large majority (78–87%) of putative haloarchaeal lipoproteins are secreted via the Tat pathway (Figure 5(e), Supplementary Table 3). In contrast, this combination is extremely rare in other archaeal species. In our *in silico *analysis, only a single exception was identified, protein AF2235 of *Archaeoglobus fulgidus* has a Tat/lipobox motif (Figure 5(e), Supplementary Table 3). 

The difference in usage of the Tat pathway and lipid-anchoring cannot be attributed to a general disparity in the level of protein secretion in these organisms. While the fraction of secreted proteins varies considerably among archaeal species, ranging from 3.6% in *S. acidocaldarius* to 10.7% in *M. hungatei*, no major differences in the range of the portion of proteins secreted are evident between the various phyla of archaea (Figure 5(f), Supplementary Table 3).

### 3.6. Development of TatLipo for Prediction of Haloarchaeal Tat Lipoproteins

Although a large number of potential Tat substrate lipoproteins were detected using the available prediction programs, our *in vivo *results show that our requirement, that at least two of the three programs used make a positive prediction, may be overly stringent (Figures 1(a) and 2(b)). This may be because these programs were trained to search for lipobox motifs within the context of Sec signal peptides, and although Tat and Sec signal peptides have significant similarities, Tat signal peptides, in addition to containing a unique twin arginine motif, are generally longer and contain a less hydrophobic h-domain than Sec signal peptides. Therefore, to identify additional potential haloarchaeal lipoproteins, we modified TatFind, which detects haloarchaeal Tat substrates, to include haloarchaeal lipobox predictions. Since the vast majority of haloarchaeal lipoproteins are secreted via the Tat pathway, this program should identify Tat lipoproteins missed by the other prediction programs used in this study.

**Figure 6:**
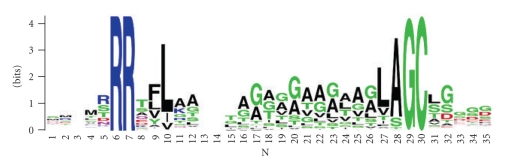
Conserved lipobox motifs of haloarchaea. Consensus motif was generated using the lipobox motifs of 400 predicted haloarchaeal Tat lipoproteins (see Supplementary Table 4). The alignment depicts the cleavage site G/A at position −1. Logos were generated using weblogo (http://weblogo.berkeley.edu/).

Although there are no confirmed Tat lipoproteins to use as a training set, the stringent lipoprotein prediction as described above, provided 484 lipoprotein candidates to be used in defining a consensus haloarchaeal lipobox motif (Figure 6). Manual alignments of the N-termini of these proteins, as described in Materials and Methods, were used to compute position-specific amino acid composition and determined that the most frequent haloarchaeal lipobox motif is LAGC and that sequence conservation increases along the motif (leucine: 60.5%, alanine: 83.5%, glycine: 98.8%, cysteine: 100%) (Figure 6 and Supplementary Table 5). Furthermore, we identified a bias in the amino acid residue composition both upstream and downstream of the LAGC sequence. A number of charged or hydrophilic amino acids (D, E, R, H, K, N, Q) are strictly forbidden in front of the lipobox motif, and at the position immediately following the conserved cysteine, leucine and serine are frequent while aspartic acid and cysteine are excluded. 

Moreover, we determined the distance between the twin arginine and the lipobox motif. The calculated number of amino acid residues between the second arginine and the conserved cysteine in the lipobox motif was in the range of 12–21.

To specifically predict Tat substrates with class II signal peptides, we incorporated the lipobox algorithm into TatFind, generating the lipoprotein prediction program TatLipo. Thus, TatLipo defines the prevalent haloarchaeal lipobox motif in Tat substrates (Figure 6). TatLipo was applied to the set of 400 predicted haloarchaeal lipoproteins that are secreted via the Tat pathway. Only three proteins in this set were not predicted by TatLipo: two of these slightly exceed the distance constraint. TatLipo predicts 113 additional lipoprotein candidates. More than two-thirds of the additional TatLipo assignments (78 of 113, 69.0%) were also predicted by one of the three other lipobox prediction programs, including Hvo_1242, which was LipoP-positive. This may indicate that many of the additional predictions are correctly called as lipoproteins. These partial confirmations are relatively evenly distributed among the three prediction programs (41 predLipo, 19 Prosite, 18 LipoP). Within the subset of lipoprotein candidates secreted via the Tat pathway, TatLipo confirmed most of the lipobox-containing proteins that were predicted by only one of the three bacterial lipoprotein prediction programs.

In conclusion, TatLipo is able to identify nearly all of the Tat-secreted lipobox proteins in halophilic archaea. While the stringent rules applied for lipobox assignment may have resulted in a number of false negatives, experimental confirmation in the future, will allow us to further improve the lipobox prediction algorithms.

## 4. Discussion

Haloarchaea transport a large fraction of their secreted proteins via the Tat pathway, possibly as an adaptation to the high salt environments they inhabit. Computational analyses of putative Tat substrates identified in several halophilic archaea have revealed that many of these precursor proteins also contain potential lipoboxes [10, 11, 22]. These findings are supported by the results obtained in the studies presented here, which also show that this predominance of SPase II cleavage sites in Tat substrates, like the extensive use of the Tat pathway to translocate secreted proteins, is a feature that is likely unique to haloarchaea. Moreover, in addition to revealing that a large portion of haloarchaeal Tat substrates appear to be lipoprotein precursors, our *in silico *analyses have also demonstrated that relatively few archaeal Sec substrates contain a genuine lipobox.

In a previous study, we determined that some haloarchaeal Tat substrates contain functional lipoboxes, with core amino acid sequences LAGC or TAGC, required for proper processing of the precursor protein and for membrane-association of the processed protein. While our *in silico* and *in vivo* data indicate that the lipobox cysteine is strictly conserved, we have determined that in addition to lipobox glycine, which is found at position −1 in 98% of the putative lipoboxes that we have identified, alanine is occasionally found at position −1. We have now shown that a haloarchaeal Tat substrate containing an alanine at lipobox position −1 requires the putative lipobox cysteine for proper processing. Moreover, a valine at the first position or a serine at the second position of the lipobox, as observed in the lipoboxes VAGC and LSAC, respectively, can also be present in haloarchaeal lipoboxes. Similar to previously tested replacement mutants, constructs lacking the lipobox cysteine were not processed. However, while previously tested mutant substrates were released into the supernatant [12], in this study, precursors containing lipoboxes in which the cysteine was replaced with a serine remained cell associated. The cell association of these mutant proteins is not that surprising since this is similar to what has been observed for the majority of bacterial lipoproteins containing a cysteine to serine lipobox substitution where unprocessed bacterial Sec substrate lipoprotein replacement mutants remain tethered to the cytoplasmic membrane via the hydrophobic stretch of the signal peptide. Analogously, it is possible that the unprocessed signal peptides of the mutant Tat substrate lipoprotein precursors investigated in this study serve a similar purpose: anchoring these unprocessed mutant proteins to the archaeal cytoplasmic membrane. It is not clear why some unprocessed lipoproteins are released into the supernatant.

We have also determined that membrane-association of an unprocessed Tat substrate containing a lipobox is not dependent on a specific Tat pore component; in fact, we have only identified a single archaeal Tat substrate that specifically requires TatAo for successful translocation (data not shown). Moreover, we have also shown that the soluble secreted protein arabinanase is secreted via Tat pores containing TatAt, demonstrating that no Tat pore is specifically dedicated to the secretion of haloarchaeal lipoproteins (Figure 3(d)).

Although our analyses clearly indicate that predicted haloarchaeal lipoprotein precursors are generally transported to the cytoplasmic membrane via the Tat pathway, the specific mechanisms involved in lipid modification and signal peptide cleavage of these substrates are currently unknown. Lnt is the evolutionarily conserved N-acyltransferase that catalyzes the acylation of the lipobox cysteine in Gram-negative bacteria. The acylation of the conserved lipobox cysteine of lipoproteins has also been confirmed in the Gram-positive bacteria *Bacillus subtilis* and *Staphylococcus aureus, *but Lnt homologs have not been identified in either of these species, indicating that an unrelated enzyme acylates lipoproteins in these organisms [31, 32]. Given that archaea also lack Lnt homologs, archaeal species that produce lipoproteins might also express a novel, archaeal-specific N-acyltransferase, or perhaps the lipoprotein acylation in archaea and Gram-positive bacteria is performed by an enzyme that is conserved between them. On the other hand, although both Gram-negative and Gram-positive bacterial species express a conserved prolipoprotein diacylglyceryl transferase, an archaeal homolog of this enzyme has not been identified, indicating that archaea are likely to express a unique enzyme that performs an analogous function. 

In addition to putative haloarchaeal Tat substrate lipoproteins, previously reported preliminary evidence has indicated that some Gram-positive bacterial lipoproteins may also be Tat substrates [14, 15]. Elucidating the mechanisms involved in modifying and processing Tat substrate lipoproteins in bacteria and archaea will almost certainly reveal important similarities and key differences in the processing of Sec substrate and Tat substrate lipoproteins.

In this study, we determined that cysteine to serine replacement mutants corresponding to two Sec substrates containing a putative lipobox are processed, and also showed that these processed mutants remain membrane-associated, perhaps by forming protein complexes with other membrane-bound proteins. The fact that the putative lipobox in these Sec substrates is conserved in homologous proteins encoded by other haloarchaeal species is interesting (data not shown). Although it is not clear whether these conserved sequences are lipoboxes, their conserved nature suggests that they may serve an important function. On the other hand, in bacteria, some lipobox cysteine to serine replacement mutants are processed by a bacterial SPase I [33]. Interestingly, all three Sec substrates investigated here contain predicted SPase I cleavage sites, as determined by Phobius (Figure 1(b)). Consistent with the hypothesis that SPase I may have processed these mutant Sec substrates, the cysteine to serine replacement mutant of the Tat substrate DsbA also contains a predicted SPase I processing site that appears to be processed, albeit inefficiently [12]. Moreover, Western blot analyses indicate that the cysteine to serine replacement mutants are less stable than the corresponding wild-type proteins, and in the case of Hvo_0494, a smaller product was detected in the protein fraction isolated from the supernatant. 

Our *in silico* data suggests that haloarchaea are unique in anchoring Tat substrates to the membrane via a lipid anchor. However, in addition, while the vast majority of the lipobox-containing proteins are secreted via the Tat pathway, haloarchaea as well as non-halophilic euryarchaeal genomes contain open reading frames that code for putative lipoproteins, which are secreted via the Sec pathway. Therefore, with regard to determining the relative importance of lipoproteins in the various archaeal phyla, further investigations of putative archaeal Sec substrate lipoproteins are necessary. These include investigation of *Hfx. volcanii* Sec substrates that contain lipoboxes, such as N-terminal amino acid sequencing of wild-type substrates and their corresponding cysteine to serine replacement mutants to determine their processing sites as well as mass spectrometry of these substrates to determine whether they are lipid modified. Furthermore, *in vivo *analyses of putative Sec substrate lipoproteins in non-haloarchaeal species, such as those predicted to be encoded by the genome of* M. mazei*, a genetically amenable methanogen, may be useful in shedding light on the significance of putative lipobox motifs in archaeal Sec substrates. 

TatLipo, the first lipoprotein prediction program primarily trained on the sequences of haloarchaeal Tat lipobox motifs, predicts a vast array of haloarchaeal Tat substrates missed by prediction programs trained solely on bacterial Sec substrates. In fact, our *in silico* analyses predicted an additional 113 haloarchaeal Tat substrate lipoproteins when results generated by TatLipo were included with those of programs trained on bacterial Sec substrates, making the utility of lipoprotein prediction programs that predict archaeal- and Tat-specific lipoproteins abundantly clear. Moreover, TatLipo detected nearly all of the proteins identified by integrating the results of three bacteria-based predictors. In addition, for the subset of Tat-secreted proteins, TatLipo confirmed two-thirds of the predictions supported by only one of these three programs, including Hvo_1242, a prediction that was supported by *in vivo* mutagenesis data. 

The inability of prediction programs trained on Sec substrates to recognize a significant portion of the Tat substrate lipoproteins may be due to key structural differences that exist between Tat and Sec signal peptides. In particular, Tat signal peptide hydrophobic stretches are less hydrophobic and their highly charged regions are longer than the corresponding regions of Sec signal peptides [28]. Future *in vivo* analyses of predicted archaeal Sec lipoproteins may help clarify the diversity of archaeal lipoproteins and may also allow the development of an archaeal Sec lipoprotein prediction program, analogous to TatLipo. Moreover, considering the fact that Gram-negative and Gram-positive bacterial Tat substrates that contain lipoboxes have also been identified, programs that will specifically determine the presence of lipoboxes in bacterial Tat substrate signal peptides will also be invaluable. TatLipo provides a solid foundation for the development of such a program.

## Supplementary Material

Supplementary Table 1: Strains and Plasmids. This table lists the strains and plasmids used in this study.
Supplementary Table 2: Primers used for PCR amplification. This table lists the primers used to amplify the constructs described in this study.
Supplementary Table 3: *in silico* secretome analysis for archaea. This table shows results of the secretome analysis for 6 halophilic archaea, 9 nonhalophilic euryarchaea, 9 crenarchaea and 3 other archaeal strains.
Supplementary Table 4: Alignment of 484 lipobox-containing proteins from halophilic archaea. The N-terminal regions of the 484 putative lipoproteins encoded by 6 halophilic archaeal genomes were aligned by introducing a gap of variable length between positions 5 and 6 after the twin-Arginine motif. The first 400 proteins are TatFind positive. The next 50 are TatFind negative but have a twin-Arginine motif. The last 34 are TatFind negative and lack a twin-Arginine motif. The results of the three lipoprotein prediction programs are indicated for each protein.
Supplementary Table 5: Position-specific amino acid frequencies. The position-specific amino acid frequencies computed for the 484 lipoproteins from 6 halophilic archaea. Amino acids in the vicinity of the lipobox motif showing a strong composition bias were used for the TatLipo algorithm, as indicated.
Supplementary Figure 1: Scheme for the prediction of archaeal lipoproteins. The figure shows a schematic representation of the assignment of secreted proteins tofour protein classes Tat/lipo, Tat/SPase I, Sec/lipo and Sec/SPase I. Data from three widely used lipoprotein prediction programs were integrated to predict the lipobox. TatFind was used to predict Tat substrates. TatFind negatives that were either predicted by Phobius or predicted to contain a lipobox are considered to be Sec substrates.
Supplementary Text: Bioinformatic Secretome Analysis. This text provides additional details concerning (a) lipoprotein prediction; (b) assignment of Tat-specific signal peptides; and (c) an evaluation of the TatLipo program and of the other lipoprotein prediction programs using the TatFind positive subset.
