# Supplementary material for: Mutational and Bioinformatic Analysis of Haloarchaeal Lipobox-Containing Proteins
Source: Archaea. 2010 Sep 16;2010:410975. doi: 10.1155/2010/410975 (PMC2945643; doi:10.1155/2010/410975)
Supplement: Supplementary file 1 — Supplementary Table 1: Strains and Plasmids. This table lists the strains and plasmids used in this study. Supplementary Table 2: Primers used for PCR amplification. This table lists the primers used to amplify the constructs described in this study. Supplementary Table 3: in silico secretome analysis for archaea. This table shows results of the secretome analysis for 6 halophilic archaea, 9 nonhalophilic euryarchaea, 9 crenarchaea and 3 other archaeal strains. Supplementary Table 4: Alignment of 484 lipobox-containing proteins from halophilic archaea. The N-terminal regions of the 484 putative lipoproteins encoded by 6 halophilic archaeal genomes were aligned by introducing a gap of variable length between positions 5 and 6 after the twin-Arginine motif. The first 400 proteins are TatFind positive. The next 50 are TatFind negative but have a twin-Arginine motif. The last 34 are TatFind negative and lack a twin-Arginine motif. The results of the three lipoprotein prediction programs are indicated for each protein. Supplementary Table 5: Position-specific amino acid frequencies. The position-specific amino acid frequencies computed for the 484 lipoproteins from 6 halophilic archaea. Amino acids in the vicinity of the lipobox motif showing a strong composition bias were used for the TatLipo algorithm, as indicated. Supplementary Figure 1: Scheme for the prediction of archaeal lipoproteins. The figure shows a schematic representation of the assignment of secreted proteins tofour protein classes Tat/lipo, Tat/SPase I, Sec/lipo and Sec/SPase I. Data from three widely used lipoprotein prediction programs were integrated to predict the lipobox. TatFind was used to predict Tat substrates. TatFind negatives that were either predicted by Phobius or predicted to contain a lipobox are considered to be Sec substrates. Supplementary Text: Bioinformatic Secretome Analysis. This text provides additional details concerning (a) lipoprotein prediction; (b) assignment of Tat-specific s [file 410975.f1.pdf]

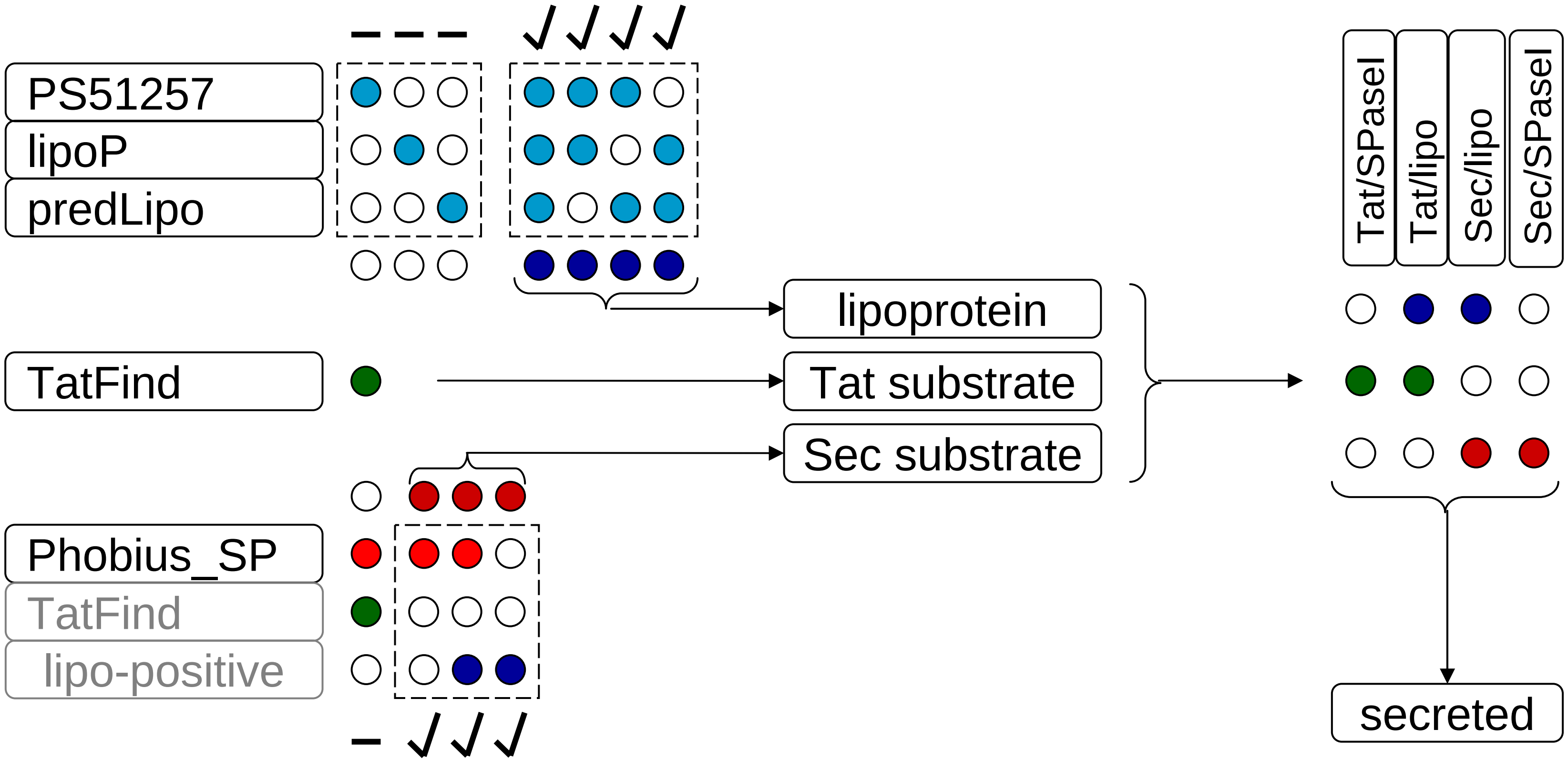

Supplementary Figure 1. Scheme for the prediction of archaeal lipoproteins. Three widely used lipoprotein prediction programs, LipoP and Prosite PS51257, predictors that were trained predominantly on Gram-negative bacterial Sec lipoproteins and PredLipo, which was trained on confirmed Gram-positive bacterial Sec lipoproteins. Lipobox positive predictions required recognition by at least two of these programs (Supplementary Table 4). TatFind was used to predict Tat substrates. Tat substrates predicted to contain a lipobox were deemed to be Tat lipoproteins, other Tat substrates were deemed to have SP with SPase I cleavage site. Predicted lipoproteins that are TatFind negative were deemed to be Sec substrates that are lipoproteins. Phobius positives that are TatFind negative and that are not predicted lipoproteins were deemed to be Sec substrates whose signal peptide is processed by SPase I.
