# Supplementary material for: Mutational and Bioinformatic Analysis of Haloarchaeal Lipobox-Containing Proteins
Source: Archaea. 2010 Sep 16;2010:410975. doi: 10.1155/2010/410975 (PMC2945643; doi:10.1155/2010/410975)
Supplement: Supplementary file 2 [file 410975.f2.pdf]

Supplementary Table 1. Strains and Plasmids

Strain/Plasmid	Relevant properties	Reference
Strain		
DH5alpha	*E. coli* Φ80d*lacZ* ΔM15 (*argF-lac*) U169 *recA1endA1 hsdR17*(r^-m^+) *supE44*$^-$ *thi*-1*gyrA96 relA1 phoA*	Invitrogen
DL739	*E. coli* MC4100 *dam-13::Tn9*	[20]
H99	*Hfx. volcanii* DS70 Δ*pyrE2* Δ*hdrB* Δ*trpA*	[21]
KD5	H99 Δ*tatAo::trpA*$^+$	[22]
KD23	H99 containing pKD13	[12]
KD26	H99 containingp pKD17	[12]
KD27	H99 containingp pKD18	[12]
KD32	H99 containingp pKD23	[12]
KD35	H99 containingp pKD26	[12]
KD37	H99 containingp pKD28	[12]
ST91	H99 containing pST10	this study
ST96	H99 containing pST11	this study
ST101	H99 containing pST12	this study
ST106	KD5 containing pST10	this study
ST107	H99 containing pST6	this study
ST111	H99 containing pST7	this study
ST114	H99 containing pST8	this study
ST118	KD5containing pST6	this study
ST126	H99 containing pST17	this study
ST128	H99 containing pST18	this study
ST135	H99 containing pST14	this study
ST137	H99 containing pST15	this study
ST139	H99 containing pST16	this study
ST167	H99 containing pST19	this study
ST168	KD5 containing pST11	this study
ST171	KD5 containing pST7	this study
ST176	KD5 containing pKD28	this study
ST177	KD5 containing pKD26	this study
ST178	KD5 containing pKD13	this study
ST179	KD5containing pKD17	this study
ST180	KD5 containing pKD23	this study
SI44	H99 containing pSI44	this study
SI45	H99 containing pSI45	this study
Plasmid		
pMLH3	(*Amp*^{r}, *Nb*^{r}, *Mev*^{r}) *H.volcanii-E.coli* shuttle vector	[18]
pST6	P_{fdx}*Hvo1242-myc* cloned into pMLH3 replacing Mev^r cassette	this study
pST7	P_{fdx}*Hvo1242C26S-myc* cloned into pMLH3 replacing Mev^r cassette	this study
pST8	P_{fdx}*Hvo1242KK-myc* cloned into pMLH3 replacing Mev^r cassette	this study
pST10	P_{fdx}*HvoB0139-myc* cloned into pMLH3 replacing Mev^r cassette	this study
pST11	P_{fdx}*HvoB0139C21S-myc* cloned into pMLH3 replacing Mev^r cassette	this study
pST12	P_{fdx}*HvoB0139KK-myc* cloned into pMLH3 replacing Mev^r cassette	this study
pST14	P_{fdx}*Hvo1808-myc* cloned into pMLH3 replacing Mev^r cassette	this study
pST15	P_{fdx}*Hvo1808C19S-myc* cloned into pMLH3 replacing Mev^r cassette	this study
pST16	P_{fdx}Δ*ssHvo1808-myc* cloned into pMLH3 replacing Mev^r cassette	this study
pST17	P_{fdx}*Hvo1580-myc* cloned into pMLH3 replacing Mev^r cassette	this study
pST18	P_{fdx}*Hvo1580C19S-myc* cloned into pMLH3 replacing Mev^r cassette	this study
pST19	P_{fdx}Δ*ssHvo1580-myc* cloned into pMLH3 replacing Mev^r cassette	this study
pKD13	P_{fdx}-*exo-arabinanase-myc* cloned into pMLH3 replacing Mev^r cassette	[12]
pKD17	P_{fdx}-*DsbA-myc* cloned into pMLH3 replacing Mev^r cassette	[12]
pKD18	P_{fdx}-*DsbAKK-myc* cloned into pMLH3 replacing Mev^r cassette	[12]
pKD23	P_{fdx}-*Ibp-myc* cloned into pMLH3 replacing Mev^r cassette	[12]
pKD26	P_{fdx}-*IbpCxS-myc* cloned into pMLH3 replacing Mev^r cassette	[12]
pKD28	P_{fdx}-*DsbAC22S-myc* cloned into pMLH3 replacing Mev^r cassette	[12]
pRV1-p*tna*	(*Amp*^{r}, *Nb*^{r}) *H.volcanii-E.coli* shuttle vector	[19]

pSI44	*Hvo0494-his* cloned into pRV1-p*tna* via *NdeI/EcoRI* restriction sites	this study
pSI45	*Hvo0494C20S-is* cloned into pRV1-p*tna* via *NdeI/EcoRI* restriction sites	this study
