# Supplementary material for: Mutational and Bioinformatic Analysis of Haloarchaeal Lipobox-Containing Proteins
Source: Archaea. 2010 Sep 16;2010:410975. doi: 10.1155/2010/410975 (PMC2945643; doi:10.1155/2010/410975)
Supplement: Supplementary file 3 [file 410975.f3.pdf]

Supplementary Table 2. Primers used for PCR amplification

Primer name	Sequence	Target sequence
703fdxOLfor	agccgaactctgcagccatgttcgacgggtctccg	1 bp downstream of Hvo1242 start codon - extension towards stop codon of Hvo1242
703mycrev	ggttccagaagcttctacagatcctcttcagagatgagttt ctgctcgccgtccagccccggaac	1 bp upstream of Hvo1242 stop codon - extension towards start codon of Hvo1242
703CSfor	gcggcgctctccgcctccctcgacagggca	67 bp downstream of Hvo1242 start codon - extension towards stop codon of Hvo1242
703Csrev	tgccctgtcgagggaggcggagagcgccgc	93 bp downstream of Hvo1242 start codon - extension towards start codon of Hvo1242
KKfor703	gggtctccgtccaagaaggagttcctgaag	10 bp downstream of Hvo1242 start codon - extension towards stop codon of Hvo1242
KKrev703	cttcaggaactccttcttggacggagaccc	39 bp downstream of Hvo1242 start codon - extension towards start codon of Hvo1242
B197fdxOLfor	agccgaactctgcagccatgctctccagacgagagg	1 bp downstream of HvoB0139 start codon - extension towards stop codon of HvoB0139
B197mycrev	ggttccagaagcttctacagatcctcttcagagatgagttt ctgctctgcgaccttcaccacccc	1 bp upstream of HvoB0139 stop codon - extension towards start codon of HvoB0139
B197CSfor	agcggggtcgctggctccggcggttcgtcg	49 bp downstream of HvoB0139 start codon - extension towards stop codon of HvoB0139
B197CSrev	cgacgaaccgccggagccagcgaccccgct	78 bp downstream of HvoB0139 start codon - extension towards start codon of HvoB0139
KKforB197	atgatgctctccaagaaggaggtactggcc	1 bp downstream of HvoB0139 start codon - extension towards stop codon of HvoB0139
KKrevB197	ggccagtacctccttcttggagagcatcat	30 bp downstream of HvoB0139 start codon - extension towards start codon of HvoB0139
134fdxOLfor	agccgaactctgcagccatgcgaaccaccggtctc	1 bp downstream of Hvo1808 start codon - extension towards stop codon of Hvo1808
134mycrev	ggttccagaagcttctacagatcctcttcagagatgagttt ctgctcgccggcgagtcccggccg	1 bp upstream of Hvo1808 stop codon - extension towards start codon of Hvo1808
fdxOLdeltaSS134	agccgaactctgcagccatgtgtgccgcccccacggct	58 bp downstream of Hvo1808 start codon - extension towards stop codon of Hvo1808
134Csfor	ttggttctcgccggctctgccgcccccacg	43 bp downstream of Hvo1808 start codon - extension towards stop codon of Hvo1808
134Csrev	cgtgggggcggcagagccggcgagaaccaa	72 bp downstream of Hvo1808 start codon - extension towards start codon of Hvo1808
ORF354fdxOLfor	agccgaactctgcagccatgtcgcccgccgccgac	1 bp downstream of Hvo1580 start codon - extension towards stop codon of Hvo1580
ORF354mycrev	ggttccagaagcttttacagatcctcctcgctgatgagctt ctgctcctcgcgggccaccgatggaa	1 bp upstream of Hvo1580 stop codon - extension towards start codon of Hvo1580
fdxOLdelss354	ccgaactctgcagccatgtgtacgtcctcatcg	73 bp downstream of Hvo1580 start codon - extension towards stop codon of Hvo1580
ORF354CSfor	gtggtactctcggggtctacgtcctcatcg	58 bp downstream of Hvo1580 start codon - extension towards stop codon of Hvo1580
ORF354CSrev	cgatgaggacgtagaccccgagagtaccac	87 bp downstream of Hvo1580 start codon - extension towards start codon of Hvo1580
ForFdx	gtaacgccagggttttcc	199 bp upstream of pyrE2 start codon on pGB70 - extension towards pyrE2 gene
RevFdx	catggctgcagagttcggct	pyrE2 start codon - extension away from pyrE2 start codon
Hvo_0494 for	agtccatatgtactccaaccgcattcc	1bp downstream of the Hvo0494 start codon – extension towards the stop codon of Hvo0494
Hvo_0494 CSfor	agtccatatgtactccaaccgcattcccgtcttacttatcg cagtcctcgtgcttctcgccgggtccgccggc	1bp downstream of the Hvo_0494 start codon – extension towards the stop codon of Hvo_0494.
Hvo_0494 ΔSS	agtccatatgtccgccggcggcgcggatgt	58 bp downstream of the Hvo_0494 start codon - extension towards the stop codon of Hvo_0494.
Hvo_0494hisrev	gaccgaattctcagtggtggtggtggtggtgcgcccgc gcttcgtcgagcc	1 bp upstream of Hvo0494 stop codon - extension towards start codon of Hvo0494
