# Supplementary material for: Mutational and Bioinformatic Analysis of Haloarchaeal Lipobox-Containing Proteins
Source: Archaea. 2010 Sep 16;2010:410975. doi: 10.1155/2010/410975 (PMC2945643; doi:10.1155/2010/410975)
Supplement: Supplementary file 4 [file 410975.f4.pdf]

Supplementary Table 3: *in silico* secretome analysis for archaea

The secretome was analyzed for several archaeal groups: 6 halophilic archaea (H), 9 non-halophilic euryarchaea (E), 9 crenarchaea (C) and 3 other archaea (OA). The organism abbreviations as given in Figure 5, the organism name, and the TaxID (http://www.ncbi.nlm.nih.gov/Taxonomy) and the total number of proteins (proteins (nr)) are given. Secreted proteins (secreted), Tat (Tat) and Sec (Sec) substrates, and lipoproteins (Lipo) were counted as described in Materials and Methods. Several combinations thereof (Tat+Lipo, Sec+Lipo) are shown. Relative contributions were computed as indicated in the column headers and are shown as % values.

archaeal group	abbreviation	organism name	taxid	proteins (nr)	secreted (nr)	Tat (nr)	Sec (nr)	Lipo (nr)	Tat+Lipo (nr)	Sec+Lipo (nr)	secreted% proteins (%)	Tat % secreted (%)	Lipo% secreted (%)	Tat+Lipo% Tat (%)	Sec+Lipo% Sec (%)	Tat+Lipo% Lipo (%)
H	Hamar	*Haloarcula marismortui* ATCC 43049	272569	4289	356	154	202	115	90	25	8.3	43.3	32.3	58.4	12.4	78.3
H	Hasal	*Halobacterium salinarum* R1	478009	2832	179	70	109	47	37	10	6.3	39.1	26.3	52.9	9.2	78.7
H	Hfvol	*Haloferax volcanii* DS2	309800	4077	297	122	175	93	77	16	7.3	41.1	31.3	63.1	9.1	82.8
H	Hqwal	*Haloquadratum walsbyi* DSM 16790	362976	2841	138	65	73	46	39	7	4.9	47.1	33.3	60	9.6	84.8
H	Namag	*Natrialba magadii* ATCC 43099	547559	4212	352	195	157	106	90	16	8.4	55.4	30.1	46.2	10.2	84.9
H	Napha	*Natronomonas pharaonis* DSM 2160	348780	2850	215	102	113	77	67	10	7.5	47.4	35.8	65.7	8.8	87
E	Arcfu	*Archaeoglobus fulgidus* DSM 4304	224325	2420	187	8	179	24	1	23	7.7	4.3	12.8	12.5	12.8	4.2
E	Mmaze	*Methanosarcina mazei* Goe1	192952	3370	214	5	209	38	0	38	6.4	2.3	17.8	-	18.2	-
E	Pyrfu	*Pyrococcus furiosus* DSM 3638	186497	2125	160	2	158	18	0	18	7.5	1.3	11.3	-	11.4	-
E	Thkod	*Thermococcus kodakarensis* KOD1	69014	2306	206	2	204	31	0	31	8.9	1	15.0	-	15.2	-
E	Methu	*Methanospirillum hungatei* JF-1	323259	3139	335	0	335	24	0	24	10.7	-	7.2	-	7.2	-

E	Metja	*Methanocaldococcus jannaschii* DSM 2661	243232	1786	97	0	97	19	0	19	5.4	-	19.6	-	19.6	-
E	Metka	*Methanopyrus kandleri* AV19	190192	1687	137	0	137	0	-	-	8.1	-	-	-	-	-
E	Picto	*Picrophilus torridus* DSM 9790	263820	1535	60	2	58	0	-	-	3.9	3.3	-	-	-	-
E	Theac	*Thermoplasma acidophilum* DSM 1728	273075	1482	60	3	57	0	-	-	4.1	5	-	-	-	-
C	Apern	*Aeropyrum pernix* K1	272557	1700	102	8	94	0	-	-	6	7.8	-	-	-	-
C	Calma	*Caldivirga maquilingensis* IC-167	397948	1963	105	7	98	0	-	-	5.4	6.7	-	-	-	-
C	Hypbu	*Hyperthermus butylicus* DSM 5456	415426	1602	100	5	95	0	-	-	6.2	5	-	-	-	-
C	Sulac	*Sulfolobus acidocaldarius* DSM 639	330779	2223	81	4	77	0	-	-	3.6	4.9	-	-	-	-
C	Sulso	*Sulfolobus solfataricus* P2	273057	2977	129	4	125	0	-	-	4.3	3.1	-	-	-	-
C	Thneu	*Thermoproteus neutrophilus* V24Sta	444157	1966	176	9	167	0	-	-	9	5.1	-	-	-	-
C	Metse	*Metallosphaera sedula* DSM 5348	399549	2256	96	4	92	1	0	1	4.3	4.2	1	-	1.1	-
C	Pyrar	*Pyrobaculum arsenaticum* DSM 13514	340102	2299	209	13	196	1	0	1	9.1	6.2	0.5	-	0.5	-
C	Ighos	*Ignicoccus hospitalis* Kin4-I	453591	1434	120	4	116	5	0	5	8.4	3.3	4.2	-	4.3	-
OA	Nequi	*Nanoarchaeum equitans* Kin4-M	228908	536	34	0	34	1	0	1	6.3	-	2.9	-	2.9	-
OA	Kocry	*Korarchaeum cryptofilum* OPF8	374847	1602	120	3	117	1	0	1	7.5	2.5	0.8	-	0.9	-
OA	Nitma	*Nitrosopumilus maritimus* SCM1	436308	1795	163	0	163	0	-	-	9.1	-	-	-	-	-
