# Supplementary material for: Mutational and Bioinformatic Analysis of Haloarchaeal Lipobox-Containing Proteins
Source: Archaea. 2010 Sep 16;2010:410975. doi: 10.1155/2010/410975 (PMC2945643; doi:10.1155/2010/410975)
Supplement: Supplementary file 5 [file 410975.f5.pdf]

Supplementary Table 4: Alignment of 484 lipobox-containing proteins form halophilic archaea

The N-terminal region of the 484 lipoproteins from 6 halophilic archaea is shown. Proteins are named by their ordered locus tag obtained upon genome annotation (code). Each of the proteins was positive in at least two of the three predictors (lipobox prediction: A: Prosite PS51257; B: lipoP; C: predLipo). The first 400 proteins are TatFind positive ("T"), the last 84 are TatFind negative ("-"). All TatFind positives and the first 50 of the TatFind negatives have a twin-Arg motif ("RR") while the last 34 TatFind negatives lack a twin-Arg motif ("-"). The twin-Arg motif and the lipobox motif were juxtaposed by introducing a gap of variable length between positions 5 and 6 after the twin-Arg motif. Spaces were added on both sides of the twin-Arg motif and of the lipobox to increase readability. If there were three or more consecutive Arg residues in the twin-Arg motif, the positioning is according to TatFind, which is based on the detection of a conserved pair of hydrophobic residues at position +2/+3. Dashes indicate the single gap of variable length introduced between position +5 and +6 after the twin-Arg motif and also the region in front of the initiator-Met.

code	N-terminal sequence	lipobox prediction	TatFind	twin-Arg
pNG2038	-------------------------------MTERT RR QLLAG------IGTSLTLA TAGC GGNSGSGGSN	A,B,C	T	RR
pNG3012	----------------------------------MK RR NLLSA--------LGTSVA LAGC STSTNSTETN	A,B,C	T	RR
pNG5085	-----------------------MTHKNIPRNSSID RR TVLGL-------LSGSLAA LAGC SGGLSDSGDG	A,B,C	T	RR
pNG6063	----------------------------------ME RR QVLKT---AGILATGGVTG LAGC SSSGNGDGGG	A,B,C	T	RR
pNG6067	-----------------------MTTRFGAPGTGLS RR EFLAA------TGATGIAS LAGC SAGGANEPAA	A,B,C	T	RR
pNG7023	-------------------------------MTNSN RR KFLKA------TGVGLLGG LAGC TRGGDSSGGD	A,B,C	T	RR
pNG7073	----------------------------------MD RR TFLAA------SGLTVAGT LAGC SAPSDNQQRD	A,B,C	T	RR
pNG7088	-------------------------------MPSTS RR TFLGM------VATGTVAS VAGC SSTCPDEGAP	A,B,C	T	RR

pNG7122	-----------------------------MNRSSVS RR QFLGA------SGAALVTG LAGC SAGEGGTSTD	A,B,C	T	RR
pNG7185	-------------------------------MHQLH RR QLLGV-------VCSGLTV LAGC GSDQSGNSSE	A,B,C	T	RR
pNG7190	-------------------------MDKADDSNGLT RR DYLSG-----GSVLLGTSI VAGC SGDGGESPST	A,B,C	T	RR
pNG7250	---------------------------MVNTNRTIS RR EMLGA------LGATGVTA LAGC SGGSDDTDTA	A,B,C	T	RR
pNG7251	---------------------------MVDKKHTRS RR ELLTA------LGAAGITG LAGC SGGGDGGDGE	A,B,C	T	RR
pNG7384	-------------------------------MVPST RR TLLHS-------VCGLATV LAGC SGVFESSGES	A,B,C	T	RR
rrnAC0147	----------------------------------MD RR SFLTA------AGATVSVS FAGC AGLGGSDESS	A,B,C	T	RR
rrnAC0158	----------------------------------MN RR QFLAS------AGLAGTTV LAGC GSLSSQSTRA	A,B,C	T	RR
rrnAC0296	------------------------------MKQSLT RR AMLGA-------LGTAVAA TAGC QSPGTGSDSG	A,B,C	T	RR
rrnAC0358	------------------------MFRNDKHSLVSK RR SFLKM------VGTAGLAG LAGC GGDSGSGDGG	A,B,C	T	RR
rrnAC0381	----------------------------MPREARVD RRR YLQA-----IGGTVATLS VAGC QSDSGDSQTL	A,B,C	T	RR
rrnAC0389	---------------------------------MET RR TFVRG------LGLAAGVA LAGC SSGGGSDSGD	A,B,C	T	RR
rrnAC0498	---------------------------------MPS RR SVLTA---------GTLVA LSGC AALSPASARL	A,B,C	T	RR
rrnAC0508	------------------------------MTNDLD RR TFIRT-----TAAVSGAGL LAGC GGSGGDGGSD	A,B,C	T	RR
rrnAC0566	------------------------------MQNTAT RRR VLGA-------LGAGVVG LSGC VSDDSSMTEQ	A,B,C	T	RR
rrnAC0576	-----------------------------MSSDTT RRR ILLGI-------GTGVTVG IAGC SGGGNGGAEE	A,B,C	T	RR
rrnAC0830	------------------------------MSSDTN RR AFLKR--TGAVTTVGLLGG LAGC STEQTGGDGG	A,B,C	T	RR
rrnAC0850	---------------------------MTQ-RR-AT RRR FLMG------CSAIASVG LAGC TGGDESTATD	A,B,C	T	RR
rrnAC0856	-------------------------------MTRFT RR GLLAA-------TVGAVGA MAGC AGGSSESESA	A,B,C	T	RR
rrnAC0899	----------------------------------MN RR EYIVA------AGVGLAGT VAGC SGQSEAGSGS	A,B,C	T	RR
rrnAC0998	-------------------------------MLRTT RRR LLSA-------LGASLSI LAGC TQFDVESGAE	A,B,C	T	RR
rrnAC1082	----------------------------------MD RR TYIGT-----IGSSMAALS LAGC SGSDGGDGNE	A,B,C	T	RR
rrnAC1892	----------------------------------MN RRR FLTL------SGVGVVGA VAGC SSEADSAESV	A,B,C	T	RR
rrnAC1944	------------------------------MEFEST RRR FMQL------AGTGATVS LAGC NALQGGDSDG	A,B,C	T	RR
rrnAC2421	--------------------------MRNPCRDARS RR QYLAA------LGGIGATA LAGC RGESGTQTEP	A,B,C	T	RR
rrnAC2469	--------------------------MNEHDTRSID RR SVIKA------AASAGLVG LAGC SGGAPDEGDG	A,B,C	T	RR
rrnAC2580	------------------------------MTNNST RRR FLKT------AGVAGVAA LAGC GGGGDGGDGG	A,B,C	T	RR
rrnAC2656	--------------------------------MSQ RRR TFISS------VGTATAIG LAGC LGGDGGDGSG	A,B,C	T	RR

rrnAC2761	-----------------------------MRNHNIT RR QLLAS------GGAAATAA LAGC AGGFGGSGQG	A,B,C	T	RR
rrnAC2848	----------------------------------M RRRR FIAT------GAGVGVGL FAGC SGSSDSGGSD	A,B,C	T	RR
rrnAC2863	----------------------------------MK RR NYIAS-------LGSGLTL LAGC ATGEGSGEGE	A,B,C	T	RR
rrnAC3028	-------------------------MRDTSQ-RR-S RR GFLGT-------LALAAVT SAGC SSLGGTQNGQ	A,B,C	T	RR
rrnAC3119	------------------------------MGRPTS RR NVIKI-------AGGALVA LAGC SDTGGSTESD	A,B,C	T	RR
rrnAC3197	----------------------------MQESSNRT RR TFIKS------AGVIGAAA LAGC SGGSDSSTED	A,B,C	T	RR
rrnAC3198	-----------------------------MTERGRT RR AFLGT------LAATTVGS VAGC QSQFNPLAST	A,B,C	T	RR
rrnAC3204	--------------------------------MDRN RR QFLGA------LTAVVTGT IAGC SGGDSGESSP	A,B,C	T	RR
rrnAC3220	-------------------------MRDQQQSGTAS RR DYLKA---AGGLAAASTVG LAGC SGSGSETGTL	A,B,C	T	RR
rrnAC3248	----------------------------------M RRR ALLAS-------VPGALAG LAGC SFGTPGSDET	A,B,C	T	RR
rrnAC3267	--------------------------------MPIQ RR EFIAA------IGTGALAT TAGC AQSGEGSGQT	A,B,C	T	RR
rrnAC3299	----------------------------MPRSSSME RR SFLKA-----TGSAAAAAA LAGC SSDSDETEGA	A,B,C	T	RR
rrnAC3315	----------------------------------M RRR QFLAG------GTVLLSAA VAGC GHPSVVLDMD	A,B,C	T	RR
rrnB0022	----------------------------------M RRR QALAL-------VTGSLSL VAGC SSDTTTDLTP	A,B,C	T	RR
rrnB0097	------------------------------MTSSYS RR TLLRT------AGVAVAGA VGGC GGDSTAQDMD	A,B,C	T	RR
rrnB0117	----------------------------------MK RR TFLAS------TGIGLTTA VAGC TTQAGSSDSP	A,B,C	T	RR
rrnB0275	----------------------------------M RRR TYIAS------GTVMASGL LAGC SSGSSESEST	A,B,C	T	RR
rrnB0319	------------------------MSASTMKQNPVD RR SVLKI------GATALAAG VAGC SQESSQSTET	A,B,C	T	RR
pNG6086	----------------------------------MN RR TFLKQ-----GTALSGGLV LSGC LGRLGFETQS	A,B,-	T	RR
pNG6087	----------------------------------MN RR DVLRA------VGGTSLVG LAGC TGLFETRSAR	A,B,-	T	RR
pNG7039	------------------------MSRYDMNREGPT RR DYMKY-----SGAVVGGSL LAGC AGQSGSEAAP	A,B,-	T	RR
pNG7040	-----------------------MTDDEKTPHERPT RR DYIKG-----AGIIAGGSL LAGC AGDAVSESTA	A,-,C	T	RR
pNG7041	-----------------------MTDPDRAACEWPT RR DIVRS-----SGALAFGGF LAGC SGESGQESSV	A,-,C	T	RR
pNG7059	----------------------------------M RRR TLLKS-----TGAAGTIVG VAGC LGGGRLGDSN	A,B,-	T	RR
pNG7234	--------------------------------MRPT RR EFLGT---------AAALS LSGC LGGTATPTLT	A,B,-	T	RR
rrnAC0082	--------------------------------MDQT RR AVLAG----------VAGL FAGC GTTSQRTTTP	A,B,-	T	RR
rrnAC0299	------------------------MSSNSNDQSELT RR EYVTC-----GGTVLAGGL LSGC TGSSGSDPSD	-,B,C	T	RR
rrnAC0460	--------------------------------MPSS RRR YLRG------CVATLGLA SAGC LGSNGYIETS	A,B,-	T	RR

rrnAC0510	------------------------------MQRPST RR QFLTG------TGVAALAI TAGC VSSGSSSEPA	A,B,-	T	RR
rrnAC0702	-------------------------------MYSDS RR GVLKK-----CIAAGSLGL SSGC LGTLGGSGDT	A,B,-	T	RR
rrnAC0906	---------------------MSDSNDMTGRNATQS RRR FLAL------GSATAATA LTGC SGILSGDDGG	-,B,C	T	RR
rrnAC0915	-------------------------MTDDNSNKRLT RR NALRI------AGAAGAAS LAGC GGSDGGDGSS	-,B,C	T	RR
rrnAC0957	--------------------------------MSRS RR TFLTT-----LAAASVTLP LAGC AGGDEQSADE	-,B,C	T	RR
rrnAC1162	--------------------------------MRIS RRR FLAT------VGAGVTLG GAGC VGDEGATAGG	A,B,-	T	RR
rrnAC1183	-------------------------------MARQT RR AVLAA-------LGSGIAA TAGC GAFGQERVDV	A,B,-	T	RR
rrnAC1260	----------------------------------MN RR DYLLA------GATLGTAG LAGC SFLAAAEAPP	A,B,-	T	RR
rrnAC1325	---------------------------------MTS RR GLLLR-----LSALTGAAG LSGC SSLLARQAES	A,B,-	T	RR
rrnAC1365	------------------------------MNRNQT RR KTLAL------LGSALSIP VAGC SSGGLFGGET	-,B,C	T	RR
rrnAC1430	----------------------------------MD RR QFLRA------AGPAAVAG LAGC LGGGSADTDY	A,B,-	T	RR
rrnAC1436	---------------------------------MRT RR TVLVL--------SAGTLC LAGC GSPGDGSNGG	-,B,C	T	RR
rrnAC1509	-------------------------MMTDSPSGGIS RRR FLTS------SGAIGTLA LAGC VQNTNSGDGS	-,B,C	T	RR
rrnAC1654	----------------------------------MN RRR YLTV-------STAALSG LAGC LGDPEYTISS	A,B,-	T	RR
rrnAC1910	---------------------------------MPQ RR DLLKT------VGIAATGL LAGC PSQGSTESNT	A,B,-	T	RR
rrnAC2010	--------------------------------MNRT RR ETLGL------VGGLAATV LGGC LAPGAGGDGG	A,B,-	T	RR
rrnAC2149	-----------------------MAHDSNGLSEFVS RR KFIAT------TGATSIAA IAGC SSGSSDSGSD	-,B,C	T	RR
rrnAC2228	----------------------------MSSGDSID RR SFLTA-----AGSAAAAAT LAGC SGDGGDGGGT	-,B,C	T	RR
rrnAC2349	--------------------------------MTME RR TVLKR-----IGGVGAATA LAGC SVQEQGNGGS	A,B,-	T	RR
rrnAC2589	-------------------------------MTLPT RR GFVVG----------CAAT LAGC GVLPEEREPI	A,B,-	T	RR
rrnAC2853	----------------------------MSTIPTAT RRR VLEA-------LGVGTAA LAGC ASAPGAKEQA	-,B,C	T	RR
rrnAC2868	----------------------------MAMDQSAS RR SFLTG------VGAAATAG LAGC TGLGFGDQTL	-,B,C	T	RR
rrnAC3111	----------------------------------MK RR KFLQT------MGVGATAV GSGC LGGGGEVVVS	A,B,-	T	RR
rrnAC3132	---------------------------MPTNGSSVN RR QLLKS---TGVAGVAGLTG LAGC SGGDGGDGGG	-,B,C	T	RR
rrnB0207	-------------------------MGSVAMHDTVS RR TVMKS------IGAAGAVG LAGC STDGGSSGNT	-,B,C	T	RR
rrnB0311	------------------------MVQSGGGYDGVS RR SVLKA---SGTALTVGTVG LAGC SSSSGASVRP	-,B,C	T	RR
OE1391R	---------------------------------MQ RRR EFLQA-----TGAALAAVG LAGC SDSGGTTDGD	A,B,C	T	RR
OE1806R	------------------------------MPTEHT RRR FLQA------TGATSIAA LAGC AGGDDGAGDG	A,B,C	T	RR

OE2157F	----------------------------------MH RR AFLAG------GTTLSVGV LAGC IGPSVSKSDY	A,B,C	T	RR
OE2171F	----------------------------MSRDTRLD RR TMLKS-----TGAAVTATL LAGC SGGGDTGDDE	A,B,C	T	RR
OE2317R	-------------------------------MADFD RR EFLKL-----AGGTVGASL VAGC SSGGGGGDTT	A,B,C	T	RR
OE3320F	---------------------------MSAMGRAPD RR TFLRS------AVAGGLAA IAGC TDRTTTGTTN	A,B,C	T	RR
OE3419F	-----------------------------MAGEAT RRR CLGHV--------AAGAAL LAGC TSHSDTSTST	A,B,C	T	RR
OE3629R	----------------------------------MG RR SFLAA-----TGAAASATT LAGC LGGTTGTPTI	A,B,C	T	RR
OE3641F	---------------------------------MTS RRR FVAA----VGSATAASLG LAGC VGDRETTTAT	A,B,C	T	RR
OE3859F	----------------------------------MH RR SFIGG------VSAAGLGI LGGC SAAVGTVAPP	A,B,C	T	RR
OE4008R	-----------------------------MRGQPVH RR SVLAL------VGGGAVSA LAGC TDTTDGDATT	A,B,C	T	RR
OE4030F	----------------------------------MH RR ALLGG------VAAAAAAS LTGC MGAQSDDQYE	A,B,C	T	RR
OE4197F	---------------------------------MPH RRR VLTA------TAAAASLS LAGC LSNDGGGDSQ	A,B,C	T	RR
OE4225F	---------------------------------MRT RR QFLAT-----TTSLTTVGL LAGC ARSPDNTDER	A,B,C	T	RR
OE4576F	----------------------------------MQ RR AFLKA-----GSAATLAGL LAGC SSPDSDATST	A,B,C	T	RR
OE4593R	---------------------------------MA RRR QILAG------GASLIAAS LAGC TSSQSPTDDG	A,B,C	T	RR
OE5273R	--------------------------------MRTT RR SFIGA------STTALLSG FAGC TSGGDDGANS	A,B,C	T	RR
OE1254R	----------------------------------M RRR TYLSL------VGSAAAAG TAGC LGVLGGDDTP	A,B,-	T	RR
OE1361F	---------------------------------MPS RR DVLRL------GAGVLAAG TAGC TDTAPNRVAA	A,B,-	T	RR
OE1597F	----------------------------------MK RR AFLAT---------AAVLT TTGC LGRTDSVSDS	A,B,-	T	RR
OE1679R	------------------------MPADDAERTTRT RR QVLAG------MGATGAAA LAGC QSTSSEDPAD	-,B,C	T	RR
OE1743R	----------------------------------MD RR TLLGR----------AVAG LTAC VSLSVGAGCL	A,B,-	T	RR
OE2175F	---------------------------MNSDQRGVP RR EFLKA-----AVAIGGASA LSAC LGRTDDPIPG	A,B,-	T	RR
OE2330R	----------------------------------MN RR AFLTA-----SAGLGSTAA LAGC LGALGFERQS	A,B,-	T	RR
OE2514F	-------------------------------MMACT RR KALAA--------VGTTLS LSGC ARIARPSSER	A,B,-	T	RR
OE3612R	-------------------------------MHSTT RR EWLGA------IGATAATG LAGC AGVGGAGQPV	A,-,C	T	RR
OE3910R	----------------------------------MD RR NFLKT---AGAAGTIGISG LSGC LGVLGGGGED	A,B,-	T	RR
OE3933F	------------------------------MSRART RRR LLSS------VALAVVAG LTGC STHRQSDTAP	A,B,-	T	RR
OE4093F	----------------------------------M RRR TVLVG------VTGALFGV GGGC LTMGADETAR	A,B,-	T	RR
OE4305R	-------------------MLLSPMTMTGDGMSNTS RRR FLKA-----TGAAALTAT VAGC SDSTSDADGS	-,B,C	T	RR

OE4357F	----------------------------------M RRR DYLRA-----VGGGATGVA AAGC LQMGGNSAET	A,B,-	T	RR
OE4485R	----------------------MHSDPDDGASGPVS RR AFVAA------TGTAGVAA LAGC ANSTDGSGGD	-,B,C	T	RR
OE4551F	-----------------------------MSDDTVS RR GFLKA----AGAATVVATS TAGC TDSGGGGDGG	-,B,C	T	RR
OE4700F	----------------------------------MH RR PFLSR------LCLGAVAA TAGC LSRRDDRDHS	A,B,-	T	RR
OE5147R	--------------------------MDEQTHTHLS RRR TLTA------SAGVLSAG LAGC ITSGDTDGDP	-,B,C	T	RR
OE5188F	-----------------------------MVDSICT RRR LLAA------VGATSVSA VAGC SSTLFGGDVE	-,B,C	T	RR
OE5404F	---------------------------MTRNNPHIS RR TALRT------VAGTALAS MAGC MDDNGSAGPN	A,B,-	T	RR
HVO_0022	----------------------------------M RRR SFLRA-----AGAGGVSAL LAGC TGTGGEQTTT	A,B,C	T	RR
HVO_0062	----------------------------MPDTNKLS RRR FLKA-----TGGAATAAA LAGC TGGDGEETTT	A,B,C	T	RR
HVO_0447	---------------------------------MAS RR EFIRT---AGITGVAGLTA LSGC TGGDDSSEAT	A,B,C	T	RR
HVO_0518	----------------------------------MN RR ALLLG-------TAGLCAS LAGC ASGGDAAEGS	A,B,C	T	RR
HVO_0530	-----------------------MSRDSQSNDNRLS RR QYVAG------AGALATMG LAGC SGGGGSNDGG	A,B,C	T	RR
HVO_0553	------------------------------MTVRYD RR TFLRT-----AAGAVGLGS LSGC VGTFGTESGE	A,B,C	T	RR
HVO_0899	---------------------------MARDSKPLN RR DVLKA---TGAVGTAGLAG LAGC TGGGDGGGGD	A,B,C	T	RR
HVO_1007	--------------------------------MNGS RR DFLAA------TGATLLGG LAGC ASAPTDDGAG	A,B,C	T	RR
HVO_1119	------------------------------MTQRVS RR SFLHA-----TAASTGIAL LAGC SSSSGDGSGG	A,B,C	T	RR
HVO_1228	--------------------------------MEYS RRR LLQT-----TGLAVAAAG LAGC NGRSSDETTT	A,B,C	T	RR
HVO_1401	----------------------------------MD RR SFVKA------AGVAGIAG LAGC TGGPSEGSED	A,B,C	T	RR
HVO_1464	------------------------MEEKSTGQEGLT RR NYIRY-----GGTALGAGM LAGC ASDSSGSDEA	A,B,C	T	RR
HVO_1624	----------------------------------MH RR TLLAA------AGTGLAAA LTGC IASAGDGADD	A,B,C	T	RR
HVO_1705	------------------------MHERDREPRGHS RRR FIAT------TAALGVGA LAGC TGGSDEETET	A,B,C	T	RR
HVO_2038	---------------------------MVNKYKRLT RR DYVKY------GAAAATAA LAGC GGQSASDPPT	A,B,C	T	RR
HVO_2141	-------------------------------MLSTT RRR TLQW------LGLGGVAS LAGC ATKSPTAAQS	A,B,C	T	RR
HVO_2149	----------------------------------M RRR AFLAS------ALTAATTL TAGC SSLGPQTEHT	A,B,C	T	RR
HVO_2286	----------------------------------MK RR KLLLG-------TTGLLTA LAGC NTITGNNKDT	A,B,C	T	RR
HVO_2375	--------------------------MTRNSDSGLS RR KFLIA------SGAAGLAG LAGC TENNTDGGGS	A,B,C	T	RR
HVO_2432	----------------------------------ME RR TFLKG-----SAGAAAAFT LAGC LGGGEGESES	A,B,C	T	RR
HVO_2607	---------------------------MTNPDARLS RR AFLGT------LGTGAAVG LAGC VGGDEGSTGD	A,B,C	T	RR

HVO_2651	-----------------------MAGRPS-RR-QLL RR GAVAV-------GVGLTGA LAGC SSLRGCSSAE	A,B,C	T	RR
HVO_2695	---------------------MVDADSRKGKRNGVS RR SFVKA-----AGASGVAVG LAGC ISTGGGDGDN	A,B,C	T	RR
HVO_A0181	--------------------------------MSLT RR TVLGS------TALLSFGL LAGC VGNGGSDDGA	A,B,C	T	RR
HVO_A0190	----------------------------------MK RR TYLRT-----LGAVGVGAA LAGC NTPDEETPTE	A,B,C	T	RR
HVO_A0192	--------------------------------MNLS RR SYLGL----------GAAV LSGC VARGGEPAGT	A,B,C	T	RR
HVO_A0299	----------------------------------MN RR NFVKF-----ASGAAAGAA LAGC AGNGGDGGSG	A,B,C	T	RR
HVO_A0362	-------------------------------MPSIS RR EYLAV------SATTVLAA TAGC QTSSCTPTDP	A,B,C	T	RR
HVO_A0380	--------------------------MVKRDKGRID RR SFLKY-----SGLAGTGIT VAGC TGNQSEETTS	A,B,C	T	RR
HVO_A0428	------------------------ MSNKSMPDTD RR KLLKT--IAAGATVGPATL LSGC TGGDAKTDVG	A,B,C	T	RR
HVO_A0477	----------------------MMAQDSKKLAGSVS RR QFLVA------SSAAGVAG LAGC SGQSNPEADA	A,B,C	T	RR
HVO_A0576	-------------------------MGKTLKSSTSN RRR FLKA-----TGASVAALT LAGC ASDDAGNGGG	A,B,C	T	RR
HVO_A0611	-------------------------------MRTKT RRR FLAT------VAGSAAVG LAGC LGGDDSSDSA	A,B,C	T	RR
HVO_A0620	-------------------------------MSLPT RR QVLRA------GGASLVAA LAGC SGEGSSSSSD	A,B,C	T	RR
HVO_B0093	--------------------------MAGKRSTNWS RR DLIKY------GAVAGTAG LAGC FDSSGQTTED	A,B,C	T	RR
HVO_B0132	---------------------------------MHT RR AVLAA------GGATALAS LSGC VGRALGALDL	A,B,C	T	RR
HVO_B0139	---------------------------------MLS RR EVLAA------GGAALVSG VAGC GGSSPAPDAT	A,B,C	T	RR
HVO_B0187	--------------------------------MVST RR SFIGG-------SAAAVAG LAGC LTTSSSEGDA	A,B,C	T	RR
HVO_B0198	---------------------------------MV RRR QILAG------SAGLFASA LAGC TAGGSADSES	A,B,C	T	RR
HVO_B0228	--------------------------MVREAITSRT RR KFIKS------AGVAGTVA LAGC AGNSDNSGDS	A,B,C	T	RR
HVO_B0358	----------------------------------MK RR TFLQL------TGLGAAGG LAGC LGSNSPPPRK	A,B,C	T	RR
HVO_0066	------------------------------MPRLRS RRR FLQL-------LGSASFG VAGC LGRDGGSSGP	A,B,-	T	RR
HVO_0154	----------------------------------MQ RR QVLAS--------LGSLAL LSGC LGGPAESPTE	A,B,-	T	RR
HVO_0564	----------------------------------MN RR TILKQ-----LAGTTAVGA LAGC VGVSETDSTQ	A,B,-	T	RR
HVO_0628	---------------------------MSQDGNDVS RRR FLTA-----AGAAAATAG LAGC SGGGGEDTTT	-,B,C	T	RR
HVO_1144	------------------------MRDHGSSNGGRT RR QFLAA------TGAAATAG LAGC ATLTASGDDF	A,-,C	T	RR
HVO_1240	---------------------------------MTS RRR FLAA------SGAVAAAG LSGC LAELGRLYTS	A,B,-	T	RR
HVO_1241	----------------------------------MK RR TYLRA------AGAGAAVG LAGC LGGGDANPNV	A,B,-	T	RR
HVO_1244	----------------------------------M RRR ALLSL------LAGAGVAG VAGC LGDGSAPAEA	A,B,-	T	RR

HVO_1245	--------------------------------MRNT RR AYLAA------TAGALTLG TAGC LGGGSGGSGN	A,B,-	T	RR
HVO_1548	----------------------------------MH RR ALLSS------AAALSLAG LSGC LADARRTTSG	A,B,-	T	RR
HVO_1597	----------------------------------ME RRR LLGL---------VAVGL SSGC LGSLPGATGP	A,B,-	T	RR
HVO_1612	------------------------------MASTTN RRR FLAL---------GVGAA IAGC LGSEPPVADD	A,B,-	T	RR
HVO_1888	--------------------------------MAIE RRR FLQA------AGVGAVLG LSGC TGNTSPPQAN	A,B,-	T	RR
HVO_1981	----------------------------------ME RR TFLKS-----VATTGALLT TAGC MGGGSESQPT	A,B,-	T	RR
HVO_2126	----------------------------------MD RR QFIAA------ASAVGATA FAGC TGGDAGGSGG	A,B,-	T	RR
HVO_2153	-----------------------MRNYVGAPGSTVS RR EFLAA------TGSVGALG LAGC AAPTNDDGNA	A,B,-	T	RR
HVO_2316	----------------------------------MN RR EMLAL------SGALAAAT IAGC TGDGQSPAET	A,B,-	T	RR
HVO_2397	-----------------------MNTNADSSDPKLS RR SVVAA------GSGLLTAG LAGC LGSGGGAGSG	-,B,C	T	RR
HVO_2799	----------------------------------MK RR ALLST------VAGSALGL AAGC LSDLDEPVET	A,B,-	T	RR
HVO_A0109	---------------------------------MRA RR NVLLG-------ATATLFG FSGC IAPFTSDGSL	A,B,-	T	RR
HVO_A0242	----------------------------------MN RR KLLLS-------LGATSLA FPGC LGKQTLNGTD	-,B,C	T	RR
HVO_A0324	----------------------------------MK RR TFVTA-------STLSVAG LSGC LGDTEYRIAD	A,B,-	T	RR
HVO_A0541	------------------------MADNDDCRSGPT RR DYVKF-----GGTVLGGGL LAGC AGGGSDATTT	-,B,C	T	RR
HVO_A0549	--------------------------MCHDCDRTVA RR TVLSA------VGVAGVAA TAGC LGGLGTDGGG	A,B,-	T	RR
HVO_A0557	------------------------MDQDLTDRATPT RR ACLKG-----GGALLAGGL LAGC AGQNDAESST	-,B,C	T	RR
HVO_A0558	----------------------------MTDSDGPT RR KFLTY-----GGSVAAGGL LAGC TGSSESDATA	-,B,C	T	RR
HVO_A0623	---------------------MRSRDARDAGDSDRT RRR FIWL------AGTAAMAG LAGC SGGGGAEPSE	A,-,C	T	RR
HVO_A0627	----------------------------------MK RR DALKT------LGIGALAA TAGC LGGFEQQSAW	A,B,-	T	RR
HVO_B0082	-------------------MGSASESVRARFGDAAS RR NFLKA------LGTAGIAG LAGC SGGGESYDGQ	-,B,C	T	RR
HVO_B0140	-------------------------------MPSYS RR DALKT-------IPALAAG LAGC ASLTGRDDSL	A,B,-	T	RR
HVO_B0144	------------------------MVDDSENSKTLT RR GCLKY-----GGTIAGSGL LAGC SGTSEPSSSS	-,B,C	T	RR
HVO_B0184	----------------------------MNEARPL RRR TVLSV------LTAASLGG LAGC LGGADDSAPD	A,B,-	T	RR
HVO_B0217	-------------------------MTDQPALTDES RRR YLKA-----IGTAGLTAG LAGC SGGGGGNGGG	-,B,C	T	RR
HVO_B0222	-------------------------------MKRTN RRR FLAA------FSSSAFAF TAGC QRPVASASER	A,B,-	T	RR
HVO_B0318	---------------------------MLDEESSIQ RR DVLSA------LGAAGVTT LAGC TGGDTGDTDD	-,B,C	T	RR
HVO_C0075	----------------------------MDSDTALD RRR YLGL------LGGSAVTG IAGC TGGSNQEGTL	-,B,C	T	RR

HQ1199A	----------------------------------MN RR ELLRV--LACTSISGGTFL TAGC SENTASFNPV	A,B,C	T	RR
HQ1310A	-------------------------MSDESRERDID RR SFVKL-----AGTATATAA LAGC GSSGGGGGAE	A,B,C	T	RR
HQ1383A	---------------------------------MSS RRR FLQG-----IGAVSTVGL VAGC TGSTNNSNNN	A,B,C	T	RR
HQ1442A	------------------------------MTSDQT RRR FISV------VGAAGITG LAGC SGGGDAGDEG	A,B,C	T	RR
HQ1471A	------------------------MSKANAGDTQVS RR KFLLT------SGAIGAAG LAGC SSSEGNSSEE	A,B,C	T	RR
HQ1486A	--------------------MFNKFDKSDLEGPKID RR TAIKL------FAAGGVSS LAGC AGESGDPGEG	A,B,C	T	RR
HQ1619A	---------------------------MVRDNDSLH RR DVLKA----AGASTVGIAG LAGC AGGGGGGDSG	A,B,C	T	RR
HQ1806A	------------------------------MSMQST RR KYIAM------IGAVGGGA MTGC LGGNTNAGGT	A,B,C	T	RR
HQ1816A	----------------------------------MK RR SYLRQSVLPLIASAGIGAV FAGC TRTESDDSRI	A,B,C	T	RR
HQ1974A	--------------------------------MEIS RRR ALES------ASVTAAVA LTGC IGESTSVSDN	A,B,C	T	RR
HQ1992A	-----------------------------MSRNVNS RR SFIKA------ASIAGIAG LAGC SGQSTSNSGG	A,B,C	T	RR
HQ2058A	----------------------------------MQ RR TLLST------LSTVGAVT IVGC IGGGDEDPQF	A,B,C	T	RR
HQ2104A	----------------------------------MN RR ELLQL------TGVSLGFI ATGC VGIDPPQDTD	A,B,C	T	RR
HQ2186A	---------------------------MFDNSGRIN RR NYIRS---VGAASVAGLAS LAGC SGDSSTDDTS	A,B,C	T	RR
HQ2192A	---------------------------MVDEKTRTS RR KFLAA------SGSLSAAA LAGC SGGGGGGGGG	A,B,C	T	RR
HQ2247A	--------------------------------MNPT RRR LLSG------LGTAAAVG LAGC SGGGDSGSTS	A,B,C	T	RR
HQ2251A	---------------------------MKQIDKPHN RR TLLSM------IIIAVSVG LAGC TGPGESEDSG	A,B,C	T	RR
HQ2450A	----------------------------------MD RR TFVKA------TGIAGIAG LAGC SGGPSGGAGN	A,B,C	T	RR
HQ2477A	------------------------------MSSNID RR TFLTT-----AGSAAAAAT LAGC SGSDDGSASD	A,B,C	T	RR
HQ2598A	--------------------------------MLTG RR GFIAA------GASAVLAG LAGC SGTLGGSASG	A,B,C	T	RR
HQ2732A	----------------------------------MD RR SYIKS-----GAGVIAGGL IAGC TGSDSESEST	A,B,C	T	RR
HQ2754A	------------------------------MYSMIQ RR KFVAG------TGAATVAA LAGC SGSSDTGDES	A,B,C	T	RR
HQ2809A	-------------------------------MSERS RR DILKG---LGAAGTAGVVS LAGC SRQSGGGSTE	A,B,C	T	RR
HQ2849A	-------------------------------MNNWS RR EFVRI------ATVGAGAG IAGC NALSPSGSIK	A,B,C	T	RR
HQ2969A	------------------------------MARDIE RR AFLKR---AGAAGVLGTVG LSGC VGGGGEGGEG	A,B,C	T	RR
HQ3009A	--------------------------------MTLT RR QALLA------TGTTLTAL LSGC GATQQEFTFS	A,B,C	T	RR
HQ3060A	--------------------------------MSWT RR GVLAS--------MAGVCG VSGC LASPDRSLPT	A,B,C	T	RR
HQ3135A	---------------------------------MPT RR TVIAA------LTTVGTAT LSGC GSFDNTADNN	A,B,C	T	RR

HQ3182A	------------------------------MTENTN RR TFLKI---AGSTGAVGLAG LAGC AGGGGGGDGT	A,B,C	T	RR
HQ3283A	------------------------------MSDGET RR EFLEA------FGVASISI LGGC QTSGDSDTQT	A,B,C	T	RR
HQ3383A	-------------------------------MIDKS RR GFLAG------VGAAATTS LAGC AGIGSGSQTL	A,B,C	T	RR
HQ3619A	-----------------------------MSDNRMS RR AVLAT------GAVGLGAS LAGC SSSGGSGGGD	A,B,C	T	RR
HQ4034A	------------------------------MDMSMN RR GVIEA------IGVGGLTV LAGC SSNSPNEQSE	A,B,C	T	RR
HQ4036A	----------------------------------MD RRR YIGI------VGTIVAGA LAGC NSNTENNAPS	A,B,C	T	RR
HQ1481A	----------------------------MVDDSEVN RRR FLRA-----TGAVAATVG VAGC SQGSDGETET	-,B,C	T	RR
HQ1855A	-----------------------------MTDSKIG RRR LLGL------TGAAVLGG LAGC NTQTQSQSNT	A,B,-	T	RR
HQ1912A	-----------------------------MLDDSLT RR ALLAS-------GVAVTLG LTGC AETDPSSTPV	-,B,C	T	RR
HQ2373A	----------------------------------MD RR NYLHA------IAGAGAFA TAGC LGSTLSFRDG	A,B,-	T	RR
HQ3303A	----------------------MSSEGADKKQRIAN RR TYLKG------LGAAGTVL LAGC SGSGGGSGGD	-,B,C	T	RR
Nmag_0005	---------------------------MKRVAPSVT RR SLLAG------ASGVGLSA LAGC SERFWSRAEN	A,B,C	T	RR
Nmag_0106	---------------------------------MVH RR NILKG------AAGVGLAS LAGC LGGDNGGVDT	A,B,C	T	RR
Nmag_0499	---------------------------MAGRDLDSA RRR FLGA------AGAAATAG IAGC VSLPTALDDR	A,B,C	T	RR
Nmag_0622	------------------------------MQTDPT RR TFLAG------SATAGIVA VAGC ASDGDGNGGE	A,B,C	T	RR
Nmag_0655	----------------------------------MN RR TALRS------AGVLATVG LAGC VDAVEEHFQG	A,B,C	T	RR
Nmag_0697	------------------------------MNGLCD RR AFLAS------AATGGMLG LAGC LSGDAEQVST	A,B,C	T	RR
Nmag_0704	------------------------------MDDRLT RRR LIAI------SGAVSAAA LAGC NDDPEDPDDP	A,B,C	T	RR
Nmag_0795	----------------------------MEGNSRYK RRR VIGL------IGTGTVVS VAGC LTDDTEDDGV	A,B,C	T	RR
Nmag_0945	------------------------------MDPSTD RRR FVQA------MGGGFAVA LAGC LSDDEENGGE	A,B,C	T	RR
Nmag_1151	--------------------------------MPLQ RR QLLAG------IGGAATLT VAGC LGDDDRGTTL	A,B,C	T	RR
Nmag_1294	----------------------------------MN RR KLLLG------SGTFVASM AAGC LQAGGTSENK	A,B,C	T	RR
Nmag_1357	-----------------------MHRPSFPS-RR-S RR AFLSG------TAVAGTAA LAGC TTLYHVLPDR	A,B,C	T	RR
Nmag_1387	------------------------------MTFRPK RR ELLMG------AGAVSLGG LAGC LSDVPGLESE	A,B,C	T	RR
Nmag_1419	----------------------------------ME RR KILLG------SGAALATA LAGC SSSETDEPSG	A,B,C	T	RR
Nmag_1438	-----------------------------MGDDSKS RR TVLKG--VGIASAAGLTTS LAGC VSQNGGSDVE	A,B,C	T	RR
Nmag_1742	-------------------------------MSDLP RRR LLAL------GGTALTGA IAGC SSSANEDDPN	A,B,C	T	RR
Nmag_1771	----------------------------------MQ RR TYLTL------TGSVGLLA VAGC LSEEQSTPKE	A,B,C	T	RR

Nmag_1801	----------------------------------MN RR QHLAQ------LGGVGLVA LSGC LDTISSDETD	A,B,C	T	RR
Nmag_1824	----------------------------------MD RR SLIHT-------AVGASFI LSGC LSRSDGNSET	A,B,C	T	RR
Nmag_1899	----------------------------------MK RR TYLTT-----TSATAVGLA LAGC MGGEESDEDP	A,B,C	T	RR
Nmag_1923	-------------------------------MSRVG RR TYLAA----AGGAVAGLAG LAGC LGDGGDSTPI	A,B,C	T	RR
Nmag_1931	----------------------------------MQ RR TVLAG-----LGSLSGLLA LSGC LSDDHGEIDE	A,B,C	T	RR
Nmag_2002	--------------------------------MELT RR KALTT-----SFSGLVMGT IAGC TDDTPEDEEE	A,B,C	T	RR
Nmag_2005	--------------------------------MDVT RR SVLKR-----TLGGAALGA LAGC LNEPDETSGT	A,B,C	T	RR
Nmag_2069	---------------------------------MPS RR SVLGA------GATAGFAT VAGC LGSNHSFGAL	A,B,C	T	RR
Nmag_2138	----------------------------------ME RRR VLAT------AGGLLPIS VAGC VTVSTESADE	A,B,C	T	RR
Nmag_2173	------------------------------MARHTT RR SVLKG---STAAAAVGTTL LAGC VTDEGNGTGD	A,B,C	T	RR
Nmag_2271	---------------------------------MIP RR KFLQC-----GVVSFSGSI IAGC SESTETSNPE	A,B,C	T	RR
Nmag_2366	-----------------------MTKRQSRSGGWSS RR TFVLA------TGAIGTSV LAGC LDGDDSDSQA	A,B,C	T	RR
Nmag_2372	---------------------------MAKFTRPVS RR GVLTS------GATAVGVA VAGC ISDDTDGAGQ	A,B,C	T	RR
Nmag_2397	------------------------------MVWNIG RR TLLSV---------TGLAL LSGC LDSGNTGETN	A,B,C	T	RR
Nmag_2411	--------------------------------MNQT RR AFLGT------TGTVGLGV VAGC LGSEDPPEPP	A,B,C	T	RR
Nmag_2669	----------------------------MASDRDQT RR QLLGL-----AGATVSTML IAGC GDTEPEDENG	A,B,C	T	RR
Nmag_2689	----------------------------------M RRR PFLVA-------ASATTVG LAGC TSAQERLGDV	A,B,C	T	RR
Nmag_2702	-----------------------------MNDSRLD RR QFLAA------AGAGLAGV VAGC SEPNNASSIE	A,B,C	T	RR
Nmag_3059	----------------------------------ME RR TYLGS---LGAAGAAGLTG LAGC LDELSGDDDQ	A,B,C	T	RR
Nmag_3090	------------------------------MVWELN RRR VLSG------VGAAGIAG LAGC IGDEEEVTEG	A,B,C	T	RR
Nmag_3154	--------------------------------MEYN RR TILQS-------LLPAVGL LAGC SFRDDVHLFE	A,B,C	T	RR
Nmag_3278	-----------------------------MADNRMT RRR LLAG------SSVAAAAG LAGC TDRLDDLRGD	A,B,C	T	RR
Nmag_3316	---------------------------------MPS RR EALLT------AGPLLSST LAGC LDTFRTSGSL	A,B,C	T	RR
Nmag_3531	----------------------------------M RRR KYLTG------ISSIATAG IAGC AGHTPTDDDQ	A,B,C	T	RR
Nmag_3965	--------------------------------MAIQ RR GFIAA------VGTGGAVG LAGC TGLGDDTGDS	A,B,C	T	RR
Nmag_4115	----------------------------------MN RR TVLTT---------AGLVA VAGC LSESSDPVTE	A,B,C	T	RR
Nmag_4271	----------------------------------MN RR SLLAM------AVPASFAA LSGC LGSNSALDDW	A,B,C	T	RR
Nmag_0096	---------------------------------MVN RR DIIKG------AGAASIAG LAGC LGGDNGGRDI	A,B,-	T	RR

Nmag_0184	---------------------------MQGANYSLD RR TLLQR--TGAATSIAGLAA IAGC IDDSNGDDDD	-,B,C	T	RR
Nmag_0242	----------------------------------MN RR GYLGA------IGAVGLTA TAGC VEELQAAADG	A,B,-	T	RR
Nmag_0327	------------------------------MTDFPT RRR VLQL------TGVGATAS LAGC TQFDIPGIGS	A,B,-	T	RR
Nmag_0438	----------------------------------MN RR TYLGA------VAGGLTAT SAGC LGDFGDSDID	A,B,-	T	RR
Nmag_0446	----------------------------MAENNPTT RR TILKV-----SGAVGAGAF LAGC ADNGNGNDEP	-,B,C	T	RR
Nmag_0460	----------------------------------M RRR TFVGT------LGGGAIAG FAGC LSREDTDDEN	A,B,-	T	RR
Nmag_0557	-------------------------------MSPS RRR FLLGA--------TGLTGA TAGC LSYVRRVSPG	A,B,-	T	RR
Nmag_0563	----------------------------------ME RR ALLTS------LCFPLFVS VSGC LAREQSPRLS	A,B,-	T	RR
Nmag_0609	--------------------------------MKYD RR TYLSS--------AIITIT LSGC TGLINSGRIR	A,B,-	T	RR
Nmag_0793	----------------------------------MN RRR ALTV------GVTTGLTA VAGC LTGMLDDESS	A,B,-	T	RR
Nmag_0856	----------------------------------MN RR QYLAS------ATGVAIVG LSGC LQILASGEIS	A,B,-	T	RR
Nmag_0963	----------------------MVDDTTMSNQYALT RR SALAS-------ASGLLVG VAGC LSGDDNEVDS	-,B,C	T	RR
Nmag_1091	--------------------------------MPST RR SLLAS-------ALVGSAA LAGC LSRVRSPSPS	A,B,-	T	RR
Nmag_1209	---------------------------------MPS RR AVLAT---------CGSAV LAGC SAVRREPSVT	A,B,-	T	RR
Nmag_1789	----------------------------MPAKNTHG RR TFLRS--TAAAGSVAALGG LAGC TGMLDGGDDT	A,B,-	T	RR
Nmag_1814	------------------------------MCPPRS RR PVLAS-------VASLSAL TAGC STNWFSNPET	A,B,-	T	RR
Nmag_1940	----------------------------------MD RR TFVHG------VGGGSVTA LAGC LTRNGENEEH	A,B,-	T	RR
Nmag_1961	----------------------------------MK RR TLLRA---TGGAVLGGSVG LAGC LDQFSDSESD	A,B,-	T	RR
Nmag_2110	-----------------------------MSQGHTD RR TMLAL-------SGAFLAG MAGC TGTAPDSEDE	-,B,C	T	RR
Nmag_2327	--------------------------------MDLN RR TMLGG------IGTASLTG LAGC LNLVGLATHE	A,B,-	T	RR
Nmag_2358	-----------------------MAPSQNLRGRGLS RR SAIAT------IGSIGALS LAGC LGGDEDGLSG	-,B,C	T	RR
Nmag_2390	----------------------------------M RRRR LLST---------GVVVA LAGC VSLPADEDET	A,B,-	T	RR
Nmag_2456	----------------------------------MH RR SVLAA------ASAVASVG IAGC LEFFEDNGED	A,B,-	T	RR
Nmag_2492	-----------------------MVENNNRYRPGIN RRR VLQG------LTAAGVIG AAGC LDSEETDETG	A,B,-	T	RR
Nmag_2581	---------------------------------MPS RRR TLAL------GATLSTTA LAGC TALRSEPEYL	A,B,-	T	RR
Nmag_2647	----------------------------------MD RR TYLST---------SAATI FAGC TELSGADEPD	A,B,-	T	RR
Nmag_2680	-------------------MN-RR-NRSANAERGTT RR TILAG------ATTAAIGL AAGC LSEDEESVPD	A,-,C	T	RR
Nmag_2733	----------------------------------MD RR QFLAA------STVGVAAT TAGC LGNALSSTDD	A,B,-	T	RR

Nmag_2739	--------------------------------MPSD RR TFLTI------CGTGATTA LAGC ADARVSARDV	A,B,-	T	RR
Nmag_2761	---------------------------------MLS RR TTLQA------TIPLFASL LSGC VSSMLPRDPP	A,B,-	T	RR
Nmag_2904	-----------------------MAHQPSGQSKLTS RR AVLSA------AGAGVTAA LAGC SDSQDEDPVR	-,B,C	T	RR
Nmag_2996	-------------------------------MTHPS RR TLLGS-------TAGLLAL TAGC IADNDDDDDD	A,B,-	T	RR
Nmag_3163	----------------------------------MD RR TWLTA------TTVGTLSA FSGC LSELADRNSS	A,B,-	T	RR
Nmag_3234	----------------------------------MH RRR VLAH------VGTGTVIG TVGC LSVPLPTGDS	A,B,-	T	RR
Nmag_3274	-------------------------------MTDSN RR SFVAA------TAAVGTLG VAGC LSTIDGWGDG	A,B,-	T	RR
Nmag_3481	---------------------------MATDPRDTT RR SLLAT------TGSLGVLG LAGC LSDDEEDDGG	A,-,C	T	RR
Nmag_3486	------------------------------MRTSFP RRR LLAT-----TGTISALAI GAGC LDDSDSESDP	A,B,-	T	RR
Nmag_3525	-----------------------------MTDRTHT RR TVLQA-----AGATSVVAL AAGC LSDDEEENGG	A,-,C	T	RR
Nmag_3601	----------------------------------MD RR EFVAA------GAVCGLGL MAGC LDDALADVTS	A,B,-	T	RR
Nmag_3725	----------------------------MLDSDHVS RR TALRL------SLASGVAI LAGC ADDNDENDVD	-,B,C	T	RR
Nmag_3851	-----------------------------MPIGKQS RRR FLTA-----TGGAAALGT VAGC LGGDDDDVVN	A,-,C	T	RR
Nmag_3944	---------------------------MMSFKDGVS RR DALKV------AGGVGLVG LAGC LGDGEQTDIT	A,B,-	T	RR
Nmag_4024	-----------------------------MASRQID RR SFLQV------AGGAGIAG LAGC LSGDVEAVSI	A,B,-	T	RR
Nmag_4050	--------------------------MEPDGNMQFN RR ELISA------LSAGGVLA LAGC ADQADGDGND	-,B,C	T	RR
Nmag_4142	----------------------------------M RRR EFIAV------GCCSVCGI SAGC LDEDDSEPVP	A,B,-	T	RR
NP0126A	----------------------------M-RR-PHS RR QFLVA------AGLTVTAA AAGC FDSASEPTGE	A,B,C	T	RR
NP0278A	----------------------------------M RRR ALLTS------CVTVAAVS ISGC FDDGESPSGG	A,B,C	T	RR
NP0322A	--------------------------------MDLS RR GVLAA----------GAVA LAGC ADLEEQADRR	A,B,C	T	RR
NP0660A	-------------------------------MPELT RRR LLAA-------AAGGIAG LAGC AAGSRESPPP	A,B,C	T	RR
NP0894A	----------------------------------M RRR TFLST------VAAGTIAA SAGC SSEELGLGLD	A,B,C	T	RR
NP0938A	--------------------------------MDST RRR MLGA------IAGSLSVV AAGC LGGDDAGDAP	A,B,C	T	RR
NP1600A	------------------------------MHTTPT RR TVVRT------AAAAAGLA VAGC LDSEPDEAPE	A,B,C	T	RR
NP1648A	------------------------------MERTV RRR TVLTV------LASSGSVL VAGC SDDDGTETPE	A,B,C	T	RR
NP1652A	------------------------------MTRKLS RR QLVSG------LAAAGAGV AAGC TDGEEGDSED	A,B,C	T	RR
NP1748A	----------------------------------M RRR TYLTA------AGGLFAAG LAGC TDEEPTGNGN	A,B,C	T	RR
NP1978A	----------------------------------MS RR SLLAA-------AGAGLAA LSGC TVLGDPPGED	A,B,C	T	RR

NP2004A	-----------------------------MRNPTVS RR KLLAS------GAAAAGIG LAGC MGGNGGSDGP	A,B,C	T	RR
NP2092A	----------------------------------MH RR TFVSG------VAAGAVAG LAGC LGGDDDAPTT	A,B,C	T	RR
NP2184A	---------------------------------MVS RR GVLAA------LAVGASSS LAGC HTGALVEVQD	A,B,C	T	RR
NP2186A	----------------------------MD-RR-VT RR GVLAA----------AGIA LAGC TDNGDDQPVR	A,B,C	T	RR
NP2288A	--------------------------MQQRLSDS RRRR TVLAA-------IGAGAAG LAGC LDGGSSNDPE	A,B,C	T	RR
NP2536A	------------------------------MSPDLT RR GYLAV------TAAAGGAA LAGC SDDVTADPDA	A,B,C	T	RR
NP2818A	-----------------------MNRDELVANKTVG RR ALLGS-------VAAFAGV VAGC SQLSPASSKE	A,B,C	T	RR
NP2860A	--------------------------------MQPS RR TFVKS-----GIGVVGLGA LAGC AEEDPDPGAE	A,B,C	T	RR
NP3028A	-------------------------------MTRYT RRR LLGS------SALAALGA FAGC TAEQPDDPEE	A,B,C	T	RR
NP3032A	-------------------------------MKRTT RRR FIAS------GAAVGAIT IAGC ADEDPEPEPE	A,B,C	T	RR
NP3040A	----------------------------------MQ RR EFLAG------GAGLTTAF TAGC ADSDRANVGI	A,B,C	T	RR
NP3142A	--------------------------MSKQTQDTWT RRR MLAV------AGAGAVVG LAGC ADESSEEPEE	A,B,C	T	RR
NP3232A	----------------------------------MD RR AFLAT------TGAAAAAS VAGC LGRGLSDEDF	A,B,C	T	RR
NP3388A	----------------------------------M RRR TYIAL-------SGAALAS LAGC SDDEGENGDG	A,B,C	T	RR
NP3578A	--------------------------------MKLT RR QALTA------FGSAGVVG MAGC LGGGNDDTEL	A,B,C	T	RR
NP3954A	------------------------------MKDIS RRR FVLGT------GATVAAAT LAGC NGNGNGNGNG	A,B,C	T	RR
NP4140A	------------------------------MTRHIN RR NVLKA------AGAASMVA LAGC NGNGNGNGNG	A,B,C	T	RR
NP4216A	---------------------------------MPS RR ELLRL--GGATLAAASAAG IAGC SEEAFEGPAD	A,B,C	T	RR
NP4616A	--------------------------------MNPS RR TVLKS-----GVGVAAAAA LAGC NGNGNGNGEE	A,B,C	T	RR
NP4692A	-------------------------------MAPP RRR QILSI------GGLGLAAA IAGC GDTAPDDDTP	A,B,C	T	RR
NP4700A	------------------------------MASDTT RR QFLIA-----ASAAATFTV VAGC SEPEEDPPGY	A,B,C	T	RR
NP4736A	---------------------------------MIS RR EFVAS------VTATGAVV VAGC GTDEDAAPEE	A,B,C	T	RR
NP4744A	-------------------------------MGTHT RRR FVRG-----TAAVAATGA LAGC NGNGNGNENG	A,B,C	T	RR
NP4750A	--------------------------------MRWT RRR VVAA------IGAATTAA VAGC SDTDEDAENG	A,B,C	T	RR
NP5018A	--------------------------------MKLH RR TALRL------AGVGVGTA VAGC LDPETDNGET	A,B,C	T	RR
NP5270A	----------------------------------M RRR TLLVS--------AAALPA VAGC SNVLPTGTND	A,B,C	T	RR
NP6072A	----------------------------------MN RR AALTV------VTTATASA LAGC GGVDIGTSGA	A,B,C	T	RR
NP6086A	----------------------------------M RRR ALLAA-------TGTACGV LAGC LDIDSRFSGS	A,B,C	T	RR

NP6254A	----------------------------------ME RR AFLMA--------TGGAAT IAGC TTGGNTEEEN	A,B,C	T	RR
NP6272A	----------------------------------MN RR AIIVA---------GGVFF LSGC GERATDMSNE	A,B,C	T	RR
NP0250A	--------------------------------MELS RR SFLAT-------SAVATAG FAGC TGDSGDPDPP	A,B,-	T	RR
NP0254A	-------------------------------MPRFD RR DYLRA------AGLAGAAA LAGC TDVFDDNGET	A,B,-	T	RR
NP0528A	----------------------------------M RRR TVLAL------AGAAGTAG LAGC LDDVLGSENG	A,B,-	T	RR
NP0544A	----------------------------MVDTSNLD RR SLLKG------VGVAGIAG LAGC TGNGDGNGNG	-,B,C	T	RR
NP0758A	-----------------------------MSDEGVD RR TFLQY--GGTAAAAAASVS VAGC NGNGDGNGNG	-,B,C	T	RR
NP0950A	---------------------------------MPT RRR VLAV--------AAAATA TAGC TALGDGENDE	A,B,-	T	RR
NP1070A	---------------------------------MRS RR TVLGA-------AAVLASG FAGC FSTEAPDDQE	A,B,-	T	RR
NP1480A	----------------------------------M RRR QVLAA----------VPVA LAGC VAAPEPGPPT	A,B,-	T	RR
NP1598A	----------------------------MTQNTNPN RR TFLKA------AGAGGAFL LAGC VGNEGEPTDE	-,B,C	T	RR
NP1654A	----------------------------------MN RR QFLAA---------GALVA AAGC LGPDDAEPPA	A,B,-	T	RR
NP1680A	---------------------------------MAT RRR FMCM-----GSTTVGLVA LSGC LGAEGAEEVD	A,B,-	T	RR
NP1858A	---------------------------------MVS RR QLLAA-------GGLASAG LAGC LDSLSDGSSP	A,B,-	T	RR
NP2396A	------------------------------METQVS RRR LLQA------GGVGAAAS FAGC GYMDTEDGGA	A,B,-	T	RR
NP2622A	-----------------------MDPQSSSQSVRMS RR SVLGA------VGAGATAA LAGC SGGQDGDPLR	-,B,C	T	RR
NP2694A	----------------------------------M RRR ELIAA---------APLVA TAGC TIGGEPPDGS	A,B,-	T	RR
NP2862A	--------------------------------MNLS RR GLLTA------GAGAATAA VAGC VDAAAPGQES	A,-,C	T	RR
NP2890A	----------------------------------MK RR DVLAG------VGATALLG TAGC LGVAGLDEHE	A,B,-	T	RR
NP3030A	--------------------------MKFRERDGLS RR EYVRA-----MVAAGGAAG LAAC LEAFDDGDTG	A,B,-	T	RR
NP3732A	--------------------------------MPPE RR DVLRL------GTVGLGGL VAGC LDNGGADFGS	A,B,-	T	RR
NP3968A	--------------------------------MELS RR DALRA-----GGGLLAAAA LSGC VEERVTRRET	A,B,-	T	RR
NP4332A	------------------------------MMPSIS RRR LLVG------GAAALGSA LAGC PSPTPGGALE	A,B,-	T	RR
NP4786A	----------------------------------MN RR SFVAG------AFGGAGIV TAGC IDIDSEDWEM	A,B,-	T	RR
NP5000A	--------------------MSQQRQTDGNDGRFTG RR AFIVG------AAATGVAA FAGC TDNGNGDGNG	A,-,C	T	RR
NP5286A	-----------------------------MSSRLVS RRR AVAV---------AATVA VAGC LTEAGRRCRG	A,B,-	T	RR
NP6022A	----------------------------MSLPPTAS RRR MLAA-----AGTALATFS LAGC SDESDDDDPE	-,B,C	T	RR
NP6104A	--------------------------MAHTPSDRVS RR SVLAT------TGAAAAFG LAGC LGGGGGDGLS	-,B,C	T	RR

rrnAC0160	----------------------------MMTERGIS RR EFAKS-----AVAIGGTAA LAAC LDRGSGTVPK	A,B,C	-	RR
rrnAC0236	--------------------------------MLLE RR HLSLA-------LVVLLIA SAGC SGLFGSETGT	A,B,C	-	RR
rrnAC0863	----------------------------------MK RR HLLLV-------AVLALVA LSGC TGFFGSEEVD	A,B,C	-	RR
rrnAC2237	-----------------------------------M RR PFALV------ILCAVALL LAGC QAPSVSPDTE	A,B,C	-	RR
pNG7056	--------------------------------MRCS RR HALRL-----IGASGVITA TAGC LNPGSLDNYA	A,B,-	-	RR
rrnAC0311	----------------------------------MY RRR VLAL--------CGLAII GAGC QGETADTETV	A,B,-	-	RR
rrnAC1046	-----------------------------------M RR LVLAV---------CLVSF LAGC GAFMTDSPPL	A,B,-	-	RR
rrnAC1739	-------------------------------MGDLS RR GFGAS---------VLAAL SAGC LGSRPDQESE	A,B,-	-	RR
rrnAC2271	----------------------------------M RRR HFVQT------LGVSALVG SAGC AEQSDDTTSA	A,B,-	-	RR
rrnAC2940	-----------------------------------M RR GVAIA-------VVCLCLL SAGC TGLFGDNAAA	A,B,-	-	RR
OE3190F	-----------------------------------M RR QLAAL-------LVVALVA VAGC AGGSAPATDD	A,B,C	-	RR
OE4563F	----------------------------------MA RR LLAVA--------VVCLVV LAGC QGGMSGDATT	A,B,C	-	RR
OE6028R	---------------------------------MSL RR LLAVF-------AVVGMVV IAGC AGGIDNGEPA	A,B,C	-	RR
OE5030R	----------------------------------MP RR VLLRS------IGVCSAVG IAGC LSQGEARSIT	A,B,-	-	RR
HVO_1806	----------------------------------MN RR LAAVF--------SAFLLV VAGC AAPVADPSAA	A,B,C	-	RR
HVO_1926	----------------------------------MQ RR TIAPI-------FLVGLVL LAGC LSAPLQTTAD	A,B,C	-	RR
HVO_2145	----------------------------MTRDTNHT RRR FTAL-----TATAALSTA LAGC SGSGGDAANA	A,B,C	-	RR
HVO_1176	----------------------------------MN RR LLVGI------AALALLLV TAGC LGGTSSVSND	A,B,-	-	RR
HVO_1477	----------------------------------MV RR ATATL-------VVVVLAV LAGC LGGGGAGAAT	A,B,-	-	RR
HVO_1673	---------------------------------MSS RR VVAVL-------ALAALVL TSGC IGFLTGEETL	A,B,-	-	RR
HVO_1802	---------------------------------MTV RRR SLGM--RGSTLLVAVLVV LAGC AAPVSPGTDG	A,B,-	-	RR
HQ1059A	----------------------------------MS RR ISRAS------IALSLLIV LAGC SGGGSLFNPN	A,B,C	-	RR
HQ2489A	----------------------------------MN RR LLLAV------CALAFLGV LSGC LGIGTGPIPA	A,B,C	-	RR
HQ3375A	-------------------------------MNVSQ RR IVMLG--------IALLVV FSGC AEVNDSIAPT	A,B,C	-	RR
HQ1196A	----------------------------------MI RR QILTK------CAVALSVG LVGC SGGGGGGSTD	A,B,-	-	RR
HQ2519A	----------------------------------MI RRR FISA-------IVAGIII IGGC TGPNDSPNTA	A,B,-	-	RR
Nmag_0702	----------------------------MNAKEIMN RR HVLSS------IGTTTGTL LAGC LTDDSGEPMA	A,B,C	-	RR
Nmag_1399	----------------------------------MY RR LLLSS---------ISLVA VSGC TSNSDKEDGT	A,B,C	-	RR

Nmag_2088	----------------------------------MY RRR LLAA-------AGLTTAV VAGC LTSDGTDGDG	A,B,C	-	RR
Nmag_2424	----------------------------------MN RR VYLAA------VGTSLSAS LAGC SSVMSVFDDE	A,B,C	-	RR
Nmag_2835	--------------------------------MYVT RR LVCVA-----------ILL TAGC VGSDDEGLDD	A,B,C	-	RR
Nmag_3079	----------------------------------MK RR HILTT------IGASLSFA TAGC LQGGSSNNEG	A,B,C	-	RR
Nmag_0572	----------------------------------MK RR LVLAT---------AGATL VSGC SSYLRAPSNS	A,B,-	-	RR
Nmag_1805	---------------------------------MVS RR HLLSS---------LGCAA LAGC TAVLPYDGTQ	A,B,-	-	RR
Nmag_2536	--------------------------------MSVP RR MMGAA----------LLGV VAGC LSIGPGAENN	A,B,-	-	RR
Nmag_2731	---------------------------------MID RR TSLVA-------VLGSILA ISGC LERISNGDTE	A,B,-	-	RR
Nmag_3155	----------------------------------MN RR LVFGS------LAVVLLIG LAGC TMFFTGISDD	A,B,-	-	RR
Nmag_3710	----------------------------------MN RR VCLRS------IAASSVVT TAGC LESLSQENDE	A,B,-	-	RR
Nmag_3769	----------------------------------MH RR VYLST------LSAAGIGS LSGC LDILATDSES	A,B,-	-	RR
NP3728A	----------------------------------M RRR VLLSL-------VSGTAAA LAGC ADGMEEANDD	A,B,C	-	RR
NP4392A	----------------------------------MK RR YYLGT---------IAAAA LAGC SELQPEEAPE	A,B,C	-	RR
NP6066A	---------------------------------MPS RR YILAC-------VGAASIA GAGC TDLSDSNGRP	A,B,C	-	RR
NP1172A	----------------------------------MD RR LLAAT-------ALAAVLL SAGC TVGYQPAADA	A,B,-	-	RR
NP4102A	----------------------------------MN RR LLAAV-------AVFVLFG LAGC TTIFGADVDD	A,B,-	-	RR
NP4728A	----------------------MSSHPRQNESKGVS RR TFALL------AGSAVTAG LAGC TDDPMENGDA	A,B,-	-	RR
NP6234A	----------------------------------MQ RR KFSTT------TGSALSIL FAGC LGGTDVPDNE	A,B,-	-	RR
rrnAC0370	-----------------------MDSGRNGGGPSVF RR DLLRS------IGAAGLAG LAGC GGSEGVTDGD	A,B,C	-	RR
OE3575R	----------------------------MRNDDCCS RR QFGTT-----LGGIGVATM IAGC SAFGLPSKKP	A,B,-	-	RR
HVO_B0280	------------------------------MRETLT RR QFGVG-------VAVATVA LAGC TDTGGGDGPV	A,B,C	-	RR
NP4850A	---------------------------MDE-RR-CT RR QVGAG------VIGGLSVL VAGC LGNGDGNSSH	A,B,C	-	RR
rrnAC1401	-----------------------------------M HR IVPAI-------GLAALVV LSGC VTATVDSTVA	A,B,C	-	-
rrnAC2054	----------------------------------MT TR LLTVL--------VVSLLV LAGC LGGGPAVGDG	A,B,C	-	-
rrnAC2239	-----------------------------------M RK ELLIA----------LTVV LAGC TVPSFGGSDH	A,B,C	-	-
rrnAC2345	---------------------------------MTA VR PLVLV-------AIAVLVS LAGC GALFGSGGEA	A,B,C	-	-
rrnB0030	--------------------------MYRFARNLCS LR GLARV------VVVVMLVT ASGC SAIGSLSSEN	A,B,C	-	-
rrnAC0024	-----------------------------------M RW LSTAA--------VVVVLL TAGC NAFVGADTAA	A,B,-	-	-

rrnAC0808	--------------------------------MASR IR VLAVV----------GLLL LAGC TGMGGSGDAG	A,B,-	-	-
rrnAC1293	-----------------------------------M RK GILTL-------LVGVLVV SSGC TGLITGETVA	A,B,-	-	-
rrnAC2079	--------------------------------- MT KR KTIFS-----SVAIALLII SGGC SGFVGGGEPV	A,B,-	-	-
rrnAC2645	--------------------------------MVRS PR VESLA--------VAALLV LAGC GGFVANGVDS	A,B,-	-	-
rrnAC2720	-----------------------------------M WR VTLAV----------GIAL IAGC SSFFPTSPTA	A,B,-	-	-
rrnAC3140	-----------------------------------M RW GVFGV--------VVVLVV ATGC SGFAGSPSQS	A,B,-	-	-
rrnAC3358	--------------------------------- MA GR IAVLL----------VLLL FAGC GAFGGAERQT	A,B,-	-	-
rrnAC3416	-----------------------------------M TR AVVAI--------VTLALV LAGC SGFVQQDSPE	A,B,-	-	-
OE3264F	----------------------------------MR PR IVLIA------ALVVSSIA LAGC SGTQSGTPTA	A,B,C	-	-
OE2121F	-----------------------------------M RA LWVAL-------AVAALAV TAGC AGFGGPQSAA	A,B,-	-	-
OE2791R	-----------------------------MVRDLHV PR TALLA------VGLAVLLV AAGC TGMPGGSSAD	A,B,-	-	-
OE3655R	-----------------------------------M VR SATIP------LVLIALLA AAGC LGSPIMEPVR	A,B,-	-	-
HVO_0494	-------------------------------- MYS NR IPVLL--------IAVLVL LAGC AGGADVAPAA	A,B,C	-	-
HVO_0807	-----------------------------------M RV SVLLA---------AALVL STGC LGGGSGTARL	A,B,C	-	-
HVO_1030	-----------------------------------M KR TGLAT------VLLALMVV LAGC AGGAGTVTET	A,B,C	-	-
HVO_1580	------------------------------ MSPAA DR GIAPL-----ALALVLLVV LSGC TSSSPSPPVV	A,B,C	-	-
HVO_1807	---------------------------------MAR FR ALPLA-------ALAALAV LSGC VGVPTADAPG	A,B,C	-	-
HVO_2535	-------------------------------MQPTA AR VLLAA----------LMVL LAGC GGGVGGDATT	A,B,C	-	-
HVO_1609	--------------------------------- MD NR LKLLS--------VALLVV LAGC TGGAADGGAG	A,B,-	-	-
HVO_1808	-------------------------------MRTTG LR AVFAA----------VLLV LAGC AAPTAAPGGD	A,B,-	-	-
HQ1800A	---------------------------------- M AR TPIIV------AGVVFLLM IAGC SAPTAPGPDD	A,B,C	-	-
Nmag_2608	-----------------------------------M RQ LAALL-------LVALLVM SAGC SAFDSSPDGD	A,B,C	-	-
Nmag_4102	-----------------------------------M KR LLLVV-------LVAAMLV LAGC ADGAGTDTDE	A,B,C	-	-
NP5284A	-----------------------------------M RI NVAAF-------AVLAVVV TAGC AGALLGEDLD	A,B,-	-	-
OE3125R	-----------------------------------M PK YSTGG--------AGSGGG GQAC ELCGSTADSL	A,B,-	-	-
HQ1883A	----------------------------------MT TK EESTN-KLIEQALTATTIA VIGC STSPGKPAHD	A,-,C	-	-
Nmag_2200	-------------------MTKRQGSNSQQVGRGLR TR VRSLP---LGLVAVLFLVV LAGC TLPASPDQFG	-,B,C	-	-
NP4668A	-----------------------------------M KL NVVGS--------GYVGTT LAAC LAEMGHNVSA	A,B,-	-	-
