# Supplementary material for: Mutational and Bioinformatic Analysis of Haloarchaeal Lipobox-Containing Proteins
Source: Archaea. 2010 Sep 16;2010:410975. doi: 10.1155/2010/410975 (PMC2945643; doi:10.1155/2010/410975)
Supplement: Supplementary file 6 [file 410975.f6.pdf]

Supplementary Table 5: position-specific amino acid frequencies

The position-specific amino acid frequency is computed for the 484 lipoproteins from 6 halophilic archaea. The row “all” is the average over the N-terminal region of the proteins, including ten residues after the conserved Cys, but excluding the initiator methionine, the twin-Arg and the lipobox motif. Position 1 is the first amino acid after the twin-Arg motif, which occurs in the 400 TatFind positives and an additional 50 TatFind negatives. The twin-Arg motif and the lipobox motif were juxtaposed by introducing a gap of variable length between positions 5 and 6 after the twin-Arg motif. Accordingly, the number of sequences is below 484 for positions 6-16. The data for positions 6-10 are not shown as the number of sequences is too low (1, 2, 11, 26, 30, respectively). The second Arg of the twin-Arg motif corresponds to pos 0 with positions immediately following (postRR). The lipobox motif is at positions 20-23 (LAGC). Amino acids relevant for the TatLipo algorithm are color-coded: (a) required (green; pos. 23 Cys); (b) frequent (blue; pos. 20 L,V,I,F; pos. 21 A; pos. 22 G; pos. 24 L,S; pos. 25 D,G;) (c) excluded (red; pos. 16-20 D,E,R,K,H,N,Q; pos. 21 C,D,E,R,K,H,N,Q,P; pos. 22 C,D,E,R,K,H; pos. 23 all amino acids except G,A,S; pos. 24 all amino acids except Cys; pos. 25 C,D; pos. 26 C) (d) tolerated (yellow, pos. 14-15 D,E,R,K,H,N,Q; pos. 20 W,Y; pos. 21 L,M,N,F,Y,W; pos. 22 S; pos. 24 E,K,Y,W; pos. 25 K,P,W) (e) normal (black).

	region	# seqs	C	D	E	G	A	V	I	L	F	Y	W	M	R	H	K	S	T	N	Q	P
all	-	-	0.5	6.6	3.9	15.8	14.9	6.9	2.6	10.3	2.4	0.8	0.2	0.8	3.1	0.7	1.5	11.1	9.9	2.9	2.1	3.1
1	postRR	484	0.2	5.6	7.0	5.4	8.7	2.3	1.4	3.7	0.4	0.6	0	0.2	16.1	1.4	4.8	9.5	19.6	3.1	8.9	1.0
2	postRR	484	0.6	0	0.4	0.2	6.0	18.8	3.9	20.2	32.4	10.7	0.6	2.1	0.2	0.2	0.2	1.0	1.9	0	0	0.4
3	postRR	484	0.2	0	0	1.2	3.7	8.9	10.1	71.5	0.6	0	0	1.0	0.2	0	0	1.0	1.0	0	0	0.4
4	postRR	484	0.2	0	0.8	10.7	30.0	4.3	1.4	5.6	0.2	0	0.2	0.6	8.5	1.2	14.9	7.2	8.7	0	5	0.4
5	postRR	484	0.6	0	0	13.0	31.8	6.6	2.9	11.0	1.0	1.7	0.2	1.4	1.7	0.2	0.4	13.2	12.8	0.2	0.8	0.4
11	preLAGC	105	1.0	0	0	21.0	26.7	3.8	8.6	6.7	0	0	0	1.0	0	0	0	8.6	21.9	0	1	0
12	preLAGC	357	1.7	0	0	22.4	23.0	13.2	7.0	10.6	1.4	0	0	0.8	0	0	0	9.0	10.9	0	0	0

13	preLAGC	424	0.7	0	0	40.1	29.0	5.2	1.9	6.1	0.5	0	0	0	0	0	0	8.3	8.3	0	0	0
14	preLAGC	448	1.1	0	0	12.7	30.6	19.9	3.8	8.7	0.2	0	0	0.2	0	0	0	6.5	15.0	0	0	1.3
15	preLAGC	469	1.1	0	0	21.7	37.3	13.0	3.2	8.3	0.6	0.2	0	0.4	0	0	0	6.2	7.5	0	0	0.4
16	preLAGC	483	0.4	0	0	28.8	28.6	10.1	0.6	14.9	2.3	0	0	0.2	0	0	0	7.7	6.0	0	0	0.4
17	preLAGC	484	1.0	0	0	7.4	21.9	12.4	6.4	24.2	1.4	0	0	1.7	0	0	0	7.0	16.1	0	0	0.4
18	preLAGC	484	0.2	0	0	13.6	33.7	18.8	4.3	10.3	2.7	0	0	0.4	0	0	0	5.8	9.9	0	0	0.2
19	preLAGC	484	0.2	0	0	27.5	27.7	10.3	3.1	13.2	1.2	0	0	1.0	0	0	0	8.5	6.8	0	0	0.4
20	LAGC	484	0	0	0	1.4	3.1	12.2	6.2	60.5	3.9	0	0	1.2	0	0	0	3.3	8.1	0	0	0
21	LAGC	484	0	0	0	1.4	83.5	0.6	0.2	0	0	0	0	0	0	0	0	11.4	2.5	0	0.2	0.2
22	LAGC	484	0	0	0	98.8	1.2	0	0	0	0	0	0	0	0	0	0	0	0	0	0	0
23	LAGC	484	100.0	0	0	0	0	0	0	0	0	0	0	0	0	0	0	0	0	0	0	0
24	postLAGC	484	0	0	0.2	6.2	11.8	6.0	2.7	26.9	0.8	0	0	1	0.2	0.2	0	23.1	15.5	2.9	2.1	0.4
25	postLAGC	484	0	13.0	4.1	42.6	8.3	1.0	0.2	0.4	0.6	0.2	0	0.2	1.2	0.2	0	17.8	6.8	1.0	2.3	0
26	postLAGC	484	0.2	11.0	6.0	22.5	3.3	3.3	2.7	7.0	3.9	0.4	0	1.2	4.3	0.6	0.4	13.0	5.6	5.6	3.5	5.4
27	postLAGC	484	0.2	12.8	6.2	24.2	6.2	3.3	1.2	5.8	4.3	0.4	0.2	1.4	1.9	0	0	14.5	6.8	3.3	2.9	4.3
28	postLAGC	484	0.2	16.9	8.9	23.8	5.8	2.1	0.6	3.1	1.0	0	0.2	0.2	2.1	0.6	0	13.6	6.0	5.2	2.5	7.2
29	postLAGC	484	0.2	14.0	11.8	21.9	8.3	1.7	0	1.4	0.6	0.8	0	0.2	2.3	0.2	0.6	16.5	8.1	3.5	2.7	5.2
30	postLAGC	484	0	17.4	10.7	16.1	6.2	2.1	0.6	2.1	1.4	0.2	0.2	0.4	2.5	0.4	0.4	14.7	6.8	7.2	2.7	7.9
31	postLAGC	484	0.2	16.7	9.7	17.4	8.9	3.9	1.2	1.2	0.4	0.2	0.2	0	1.9	0	0.4	13.4	11.0	3.1	2.3	7.9
32	postLAGC	484	0.2	14.7	11.8	15.1	6.6	3.3	1.4	1.4	0.6	0.6	0	0.6	2.1	1.0	0.6	11.4	11.2	5.6	2.9	8.9
33	postLAGC	484	0	14.0	12.4	14.5	8.7	2.9	1.4	3.9	0.6	0.6	0.4	0.2	2.7	0.6	0.8	10.1	13.2	4.5	1.9	6.4
