# Supplementary material for: Mutational and Bioinformatic Analysis of Haloarchaeal Lipobox-Containing Proteins
Source: Archaea. 2010 Sep 16;2010:410975. doi: 10.1155/2010/410975 (PMC2945643; doi:10.1155/2010/410975)
Supplement: Supplementary file 7 [file 410975.f7.pdf]

Supplementary Text: Bioinformatic Secretome Analysis

Lipoprotein prediction.

Three lipoprotein prediction programs were used in the first phase of this study: Prosite PS51257, lipoP, and predLipo. Initially, we analyzed the correlations between the predictions of these three programs. A total of 635 proteins were positive with at least one of the predictors. 42.7% of these lipoprotein candidates were predicted by all three programs, while 23.8% were predicted by a single program.

In order to avoid false positive assignments, we considered a protein lipobox-positive only when it was identified by at least two of the prediction programs. In the following, these are referred to as "haloarchaeal lipoproteins". It should, however, be noted that this definition of "haloarchaeal lipoproteins" is operational. We cannot be certain that all of the predictions are real lipoproteins, nor can we exclude that some of the unique predictions actually do contain a lipobox, which is recognized *in vivo*.

Of the 484 proteins thus selected, 56% were predicted by all 3 programs; the other 44% were predicted by two of the three programs. The predictions made by each program are shown in Supplementary Table 4.

# of methods	lipoproteins	%	raw predictions	%
3	271	56.0%	271	42.7%
2	213	44.0%	213	33.5%
1	-	-	151	23.8%
total	484	100.0%	635	100.0%

We then analyzed the performance of the three programs on the set of 484 lipobox-positives encoded by the six haloarchaeal genomes. Only a few haloarchaeal lipoproteins were missed by lipoP, which made a relatively small set of 38 unique predictions not supported by either Prosite or predLipo. Prosite predicted four times as many false negatives as lipoP, and the number of unique predictions (61) was also significantly higher. More than

30% of the haloarchaeal lipoproteins were not predicted by predLipo, which made 52 unique predictions.

method	trained on organism group	positive on lipoproteins	negative on lipoproteins	unique positive prediction	positive (%)	negative (%)
Prosite	gram-negative bacteria	435	49	61	89.9%	10.1%
lipoP	gram-negative bacteria	472	12	38	97.5%	2.5%
predLipo	gram-positive bacteria	332	152	52	68.6%	31.4%
total lipoproteins		484			100.0%	

Assignment of Tat-specific signal peptides:

To predict Tat substrates, we used TatFind, which applies stringent criteria and thus is more likely to generate false negative than false positives

As stated in Materials and Methods, many TatFind positives are also predicted by Phobius to have a Sec signal peptide sequence (455 of 708, 64.2%). For TatFind positives, we discarded Phobius predictions of Sec signal peptides.

We generated a manual alignment of all haloarchaeal lipoproteins (Supplementary Table 4). In addition to the 400 TatFind positives, 50 additional lipoproteins have appropriately spaced Arg residue pair. Further inspection indicated that these failed to be predicted because TatFind does not allow one or more amino acids that occur in the vicinity of the pair of arginines (at positions +1, +4, +5 and/or +6).

Currently, we have designated these additional 50 proteins to be Sec substrates. Future studies will determine whether TatFind constraints should be modified.

We want to emphasize that we cannot exclude the possibility that we have underestimated the percentage of secreted proteins that are Tat substrates. Although our

analysis already points to extensive use of the Tat pathway in halophiles, our estimate may be conservative if some of our TatFind negatives having paired Arg residues in their C-terminal charged region are secreted via this pathway.

	proteins	%
TatFind	400	82.6%
additional sequences with pair of arginines	50	10.3%
total lipoproteins	484	100.0%

Evaluation of TatLipo.

We manually aligned the 484 lipoproteins from the six haloarchaeal genomes in order to compute position-specific amino acid composition data as described in Materials and Methods. We re-analyzed the performance of the different lipoprotein prediction tools on the subset of 400 lipoproteins that are TatFind-positive. These data were correlated with those obtained with TatLipo.

In general, the performance of the different tools is similar to those observed for the complete set of lipoproteins. An exception is predLipo, which performs better on the TatFind-positive subset.

TatLipo detects all but three haloarchaeal lipoproteins, two of which slightly exceed the distance constraint.

For Prosite and lipoP, the number of unique positive predictions is largely reduced (from 61 to 20 and from 38 to 20, respectively). TatLipo supports nearly all of these unique positive predictions. The reduction in unique positive predictions is less extensive for predLipo (52 to 41). The remaining 41 proteins are all supported by TatLipo.

On the other hand, TatLipo predicts an additional 113 candidates. Of these unique TatLipo positives, 78 (69%) are supported by one of the other three predictors, with each of these confirming a subset of the TatLipo predictions. Thus, this is not a casual correlation due to algorithmic similarities with one of the other predictors. We are confident that a large

number of the additional TatLipo predictions are true positives. When only those 78 are counted, which are supported by one of the three other programs, the number of lipoproteins increased by 20% (78 of 400).

method	trained on organism group	positive on Tat / lipoproteins	negative on Tat / lipoproteins	unique positive prediction	supported by TatLipo / by other predictors	positive (%)	negative (%)
Prosite	gram-negative bacteria	352	48	20	19	88.0%	12.0%
lipoP	gram-negative bacteria	389	11	20	18	97.2%	2.8%
predLipo	gram-positive bacteria	290	110	41	41	72.5%	27.5%
TatLipo	halophilic archaea	397	3	113	78	99.2%	0.8%
total lipoproteins		400				100.0%	
